# The transcription factor PDR-1 is a multi-functional regulator and key component of pectin deconstruction and catabolism in *Neurospora crassa*

**DOI:** 10.1186/s13068-017-0807-z

**Published:** 2017-06-12

**Authors:** Nils Thieme, Vincent W. Wu, Axel Dietschmann, Asaf A. Salamov, Mei Wang, Jenifer Johnson, Vasanth R. Singan, Igor V. Grigoriev, N. Louise Glass, Chris R. Somerville, J. Philipp Benz

**Affiliations:** 1 HFM, TUM School of Life Sciences Weihenstephan, Technical University of Munich, Freising, Germany; 2 Department of Plant and Microbial Biology, University of California, Berkeley, Berkeley, CA USA; 3 Energy Biosciences Institute, University of California, Berkeley, Berkeley, CA USA; 4 Department of Infection Biology, Institute for Clinical Microbiology, Immunology and Hygiene, Universitätsklinikum Erlangen and Friedrich-Alexander Universität, Erlangen-Nuremberg, Germany; 5 US Department of Energy Joint Genome Institute (JGI), Walnut Creek, CA USA; 6 Environmental Genomics and System Biology, Lawrence Berkeley National Laboratory, Berkeley, CA USA

**Keywords:** Pectin degradation, l-rhamnose catabolism, d-galacturonic acid catabolism, Gene regulation, Zn_2_Cys_6_ transcription factor, *Neurospora crassa*, Biorefinery

## Abstract

**Background:**

Pectin is an abundant component in many fruit and vegetable wastes and could therefore be an excellent resource for biorefinery, but is currently underutilized. Fungal pectinases already play a crucial role for industrial purposes, such as for foodstuff processing. However, the regulation of pectinase gene expression is still poorly understood. For an optimal utilization of plant biomass for biorefinery and biofuel production, a detailed analysis of the underlying regulatory mechanisms is warranted. In this study, we applied the genetic resources of the filamentous ascomycete species *Neurospora crassa* to screen for transcription factors that play a major role in pectinase induction.

**Results:**

The pectin degradation regulator-1 (PDR-1) was identified through a transcription factor mutant screen in *N. crassa*. The Δ*pdr*-*1* mutant exhibited a severe growth defect on pectin and all tested pectin-related poly- and monosaccharides. Biochemical as well as transcriptional analyses of WT and the Δ*pdr*-*1* mutant revealed that while PDR-1-mediated gene induction was dependent on the presence of l-rhamnose, it also strongly affected the degradation of the homogalacturonan backbone. The expression of the endo-polygalacturonase *gh28*-*1* was greatly reduced in the Δ*pdr*-*1* mutant, while the expression levels of all pectate lyase genes increased. Moreover, a *pdr*-*1* overexpression strain displayed substantially increased pectinase production. Promoter analysis of the PDR-1 regulon allowed refinement of the putative PDR-1 DNA-binding motif.

**Conclusions:**

PDR-1 is highly conserved in filamentous ascomycete fungi and is present in many pathogenic and industrially important fungi. Our data demonstrate that the function of PDR-1 in *N. crassa* combines features of two recently described transcription factors in *Aspergillus niger* (RhaR) and *Botrytis cinerea* (GaaR). The results presented in this study contribute to a broader understanding of how pectin degradation is orchestrated in filamentous fungi and how it could be manipulated for optimized pectinase production.

**Electronic supplementary material:**

The online version of this article (doi:10.1186/s13068-017-0807-z) contains supplementary material, which is available to authorized users.

## Background

Plant pathogenic and saprotrophic fungi have developed elaborate mechanisms to depolymerize plant cell wall polysaccharides and subsequently catabolize liberated carbohydrates. This ability makes them not only central players in the global carbon cycle, but also major pests in agriculture as well as useful for biotechnological applications, such as for food and beverage processing or for biorefineries (see e.g., [1, 2]). While secondary plant cell walls are composed predominantly of cellulose and hemicelluloses, fruits and leaves have a high content of primary cell walls, which are rich in pectin [3]. Pectins, the most structurally heterogeneous type of plant polysaccharides, are able to form highly dynamic gelling structures and are the major polysaccharides of the middle lamella,  which is the shared interface and connecting layer between plant cells. They thus play an integral role in plant cell growth and morphogenesis [4, 5]. At the molecular scale, crosslinking of pectin to hemicelluloses, lignin, and structural proteins within the plant cell wall network affects pore size and cell wall flexibility [6], limiting the free diffusion of hydrolytic enzymes and thus contributing to biomass recalcitrance [7].

The production of pectinases by plants during leaf abscission [8] or by phytopathogenic fungi during host colonization [9–11] highlights the importance of targeted degradation of pectin during maceration processes. The adaptation of this process for artificial maceration is routinely used in food and wine production for clarification, stabilization, and reduction of viscosity [12, 13]. Moreover, in industrial plant biomass fermentations, pectin degradation has been shown to lower the viscosity of the slurries [14]. Finally, the constituent sugars of pectin are a potentially valuable feedstock for biomaterials or biofuels, if efficiently liberated and converted [15, 16].

One of the hallmarks of pectin is its high content of charged sugars. d-Galacturonic acid (d-GalA) is the most abundant backbone monosaccharide and can comprise up to 70% of pectin [6]. When unsubstituted by other sugar moieties, the polymer of α-1,4-linked GalA is called homogalacturonan (HG). Xylogalacturonans (XGA) are HG polymers decorated by d-xylose (d-Xyl) residues, while the pectin domain called rhamnogalacturonan-II (RG-II) is the most structurally complex HG derivative, with four conserved side chains composed of 12 different monosaccharide constituents [17]. The second most abundant pectin domain is rhamnogalacturonan-I (RG-I) which has a backbone of alternating d-GalA and l-rhamnose (l-Rha) residues, of which about half are decorated with side chains mostly made of l-arabinose (l-Ara), d-galactose (d-Gal), or both (arabinogalactan). Adding to the complexity of pectin are associated glycoproteins [18] or structural proteoglycans [19], as well as the fact that the domain ratio and its modifications are highly variable [6, 20].

It has been shown previously that *Neurospora crassa* (*N. crassa*) grows well on pectin and induces a very specific set of genes that differs quite substantially from those being upregulated on cellulose and xylan [21], suggesting the existence of an independent regulatory system. In particular, a set of eight genes coding for enzymes that degrade the HG and RG-I backbone as well as a number of genes encoding enzymes with the potential to depolymerize the RG-I arabinogalactan side chains are strongly induced. Among the HG-active enzymes are one endo- and one exo-polygalacturonase (GH28-1 and GH28-2, respectively), the pectin methylesterase CE8-1, and two putative pectate lyases: PLY-1 (family PL1) and PLY-2 (family PL3). It is noteworthy that pectin lyase activity has been detected in *N. crassa*, indicating that either PLY-1 or PLY-2 can act on methyl-esterified pectin [22]. The RG-I backbone is broken down in *N. crassa* by the combined action of three different enzymes: the rhamnogalacturonan lyase ASD-1 (family PL4), the putative rhamnogalacturonan acetylesterase CE12-1, and the intracellular putative unsaturated rhamnogalacturonyl hydrolase GH105-1. Regarding the RG-I and XGA side chains, *N. crassa* secretes five putative arabinosidases/α-l-arabinofuranosidases (GH43-4, GH43-6, GH43-7, GH51-1, and GH54-1), one putative endo-β-1,6-galactanase (GH5-5), the putative arabinogalactan endo-β-1,4-galactosidase GH53-1, two β-galactosidases (GH35-1 and GH35-2), two putative β-1,4-xylosidases (GH3-7 and GH43-3), the β-glucuronidase GH79-1, and the feruloyl esterase FAE-1 [21]. More members of the respective families are encoded in the genome [23] but have not been found to be secreted or induced strongly on pectin. Therefore, with the exception of an α-l-rhamnosidase (GH78), *N. crassa* has a complete, but non-redundant, set of enzymes to depolymerize all major pectic domains.

The complexity of pectin as substrate requires fungi to fine-tune their hydrolytic potential in a similarly complex manner, which is thought to require a multi-faceted regulation system [24]. Previous studies, mostly in the highly pectinolytic fungi *Aspergillus niger* (*A. niger*) and *Botrytis cinerea (* *B. cinerea*), have shown that individual sugars liberated from pectin by secreted enzymes, such as d-GalA, l-Ara, l-Rha and d-Gal, or their respective metabolic derivatives have an inducing potential for the corresponding gene products [25–27]. Recently, transcription factors (TFs) that are conserved across fungi and that mediate the response to some of these monosaccharides have been identified: TRC1 and RhaR for l-Rha in *Pichia stipitis* (*P. stipitis*) and *A. niger*, respectively [28, 29], GaaR for d-GalA in *B. cinerea* [30] and Ara1 for l-Ara in *Magnaporthe oryzae* (*M. oryzae*) [31]. While identified in different fungi, these TFs have in common that they are responsible for regulating a subset of genes involved in the degradation of a particular pectin domain or pectic polysaccharide: HG for GaaR, the RG-I backbone for RhaR, and the arabinan side chains of RG-I (or hemicellulosic arabinan) for Ara1. Intriguingly, the RhaR, GaaR, and Ara1 deletion mutants do not display a major growth phenotype on complex pectin. A more specialized TF function might be advantageous in fungi with expanded pectinolytic gene sets that need to be coordinated, such as found in *A. niger* and *B. cinerea*. *N*. *crassa* on the other hand, is a generalist saprotroph, which grows robustly on a wide array of carbon sources using a rather small, non-redundant lignocellulolytic gene complement, and has been instrumental in the last few years to study transcriptional regulation in filamentous fungi (see [32, 33] for a review). Therefore, we reasoned that using *N. crassa* with its genetic resources might allow us to identify a TF with a broader function in pectin degradation with a potential use in biotechnological applications.

In the present study, we performed a screen using the *N. crassa* deletion strain set [34] to identify TFs with a major growth phenotype on pectin. The TF candidate with the strongest and most specific growth reduction on pectin was designated pectin degradation regulator-1 (*pdr*-*1*). We show that PDR-1 is responsive to l-Rha, similar to RhaR in *A. niger*, and that its activity might be influenced by the presence of this sugar. However, PDR-1 has a novel regulatory role, combining functions of RhaR and GaaR by influencing gene expression associated with RG-I and HG catabolism.

## Results

### Identification of NCU09033 as putative pectin degradation regulator

Three enzymes, namely the endo- and exo-polygalacturonases GH28-1 (endo-PG), GH28-2 (exo-PG), and the pectin methylesterase (PME) CE8-1 have previously been shown to be essential for growth of *N. crassa* on pectin [21], indicating that HG/d-GalA could be the main carbon source for *N. crassa* in this polysaccharide. Therefore, we used the transcription factor (TF) deletion strain set of the Fungal Genetics Stock Center [35] together with the wild-type (WT) strain and the Δ*gh28*-*2* mutant (as control) to search for TF mutants that showed atypical growth on pectin as a sole carbon source [21]. Twelve TF deletion strains showed a substantial growth defect on pectin and were used for a secondary screen on medium containing either 2% sucrose, 1% cellulose, or 1% xylan as sole carbon sources. Of these 12 mutant strains, only the NCU09033 deletion strain showed a severe growth phenotype that was highly specific for pectin (data not shown). The NCU09033 locus was therefore named *pectin degradation regulator*-*1* (*pdr*-*1*).

The 971 amino acid long TF PDR-1 contains two conserved domains, a GAL4-like Zn(II)_2_Cys_6_ binuclear cluster DNA-binding domain and a fungal transcription factor regulatory middle homology region (fungal TF MHR), similar to its orthologs in *A. niger*, *Aspergillus nidulans* (*A. nidulans*), and *P. stipitis* (Additional file 1: Figure S1). The PDR-1 amino acid sequence also contains a predicted nuclear localization signal (NLS) and a predicted nuclear export signal (NES). Sequence alignments of PDR-1 to RhaR of *A. niger* and *A. nidulans* showed an identity of 47 and 48% between the full-length sequences, respectively, or 60 and 61% when just the fungal TF MHRs were compared. However, PDR-1 shares only 28% sequence identity with TRC1, and the fungal TF MHR of both proteins only shows 23% identity.

**Fig. 1:**
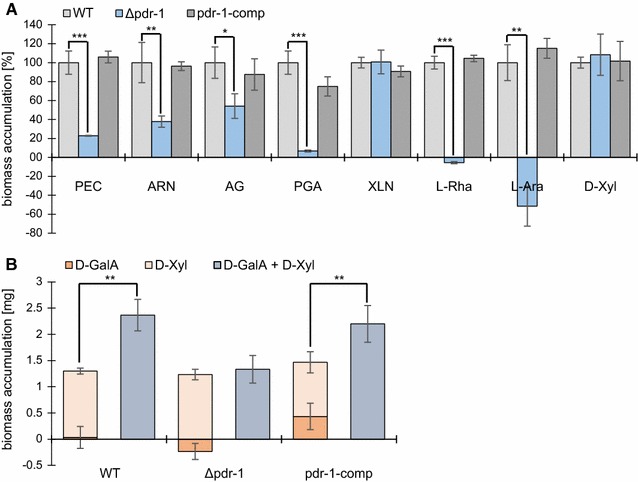
Biomass accumulation of the WT, Δ*pdr*-*1*, and pdr-1-comp strain. *PEC* pectin, *ARN* arabinan, *AG* arabinogalactan, *PGA* polygalacturonic acid, *XLN* xylan. *Error bars* represent standard deviation (*n* = 3). **A** The biomass of *N. crassa* WT, Δ*pdr*-*1*, and pdr-1-comp was determined. *S*trains were grown for 3 days in 3 ml cultures with 1% polysaccharide as carbon source. Alternatively, the strains were pre-incubated for 16 h in medium containing 2% sucrose, then washed, transferred to a 2 mM monosaccharide medium and incubated for additional 3 days with regular medium switches. After incubation, the biomass was determined as dry weight. The samples were normalized to WT biomass. Significance was determined by an independent two-sample *t*-test of WT against Δ*pdr*-*1* or pdr-1-comp with *p < 0.05, **p < 0.01, and ***p < 0.001. **B** Same strains as mentioned under **A** were grown on either 2 mM d-GalA, 0.5 mM d-Xyl or 2 mM d-GalA and 0.5 mM d-Xyl. Significance was determined by an independent two-sample *t*-test of 0.5 mM d-Xyl against 2 mM d-GalA and 0.5 mM d-Xyl with **p < 0.01

To confirm that the inability to grow on pectin was due to the deletion of *pdr*-*1*, the *pdr*-*1* gene, under control of its native promoter and terminator, was transformed into the Δ*pdr*-*1* strain. This strain, named pdr-1-comp, showed a WT-like phenotype when grown on pectin, indicating a functional complementation of the NCU09033 deletion mutation (Fig. 1A; Additional file 1: Figure S2A). An analysis of the secreted proteins of WT, Δ*pdr*-*1*, and pdr-1-comp strains after incubation on pectin showed that secretion rates were restored in the pdr-1-comp strain (Additional file 1: Figure S2B).

### PDR-1 is a regulator of pectin depolymerization and catabolism

The observed growth defect of the *N. crassa* Δ*pdr*-*1* strain on pectin (Additional file 1: Figure S2A) suggested that PDR-1 might regulate a broader range of genes related to pectin degradation than the previously described PDR-1 homologs RhaR. To test this hypothesis, the Δ*pdr*-*1* mutant as well as the *N. crassa* WT and pdr-1-comp strains were grown on several pectic carbon sources [pectin, arabinan, arabinogalactan, polygalacturonic acid (PGA)] and xylan as a hemicellulose control (Fig. 1A). The dry weight of the strains was determined after 3–7 days (depending on carbon source). The Δ*pdr*-*1* mutant strain exhibited a significant growth defect on all pectin-related carbon sources, including the ones that are side-chain-related. Growth of the pdr-1-comp strain was indistinguishable from the WT, confirming that the Δ*pdr*-*1* mutant phenotype is the result of a lack of PDR-1.

In addition to growth experiments on polysaccharides, the WT, Δ*pdr*-*1*, and pdr-1-comp strains were shifted to the monosaccharides l-Rha and l-Ara along with d-Xyl as control following a 16 h pre-incubation on 2% sucrose (Fig. 1A). After an additional 2 days of growth, the Δ*pdr*-*1* strain did not accumulate any biomass on l-Rha and l-Ara like the WT and pdr-1-comp strains, but rather lost weight. These results indicate that *pdr*-*1* is necessary for l-Rha and l-Ara catabolism.

When we tested the growth of the WT, Δ*pdr*-*1*, and pdr-1-comp strains on d-GalA as the only carbon source, even the WT strain grew poorly (data not shown). d-GalA is more oxidized than neutral monosaccharides, and an increased amount of reduction equivalents is necessary for its catabolism, making this process energetically expensive [16, 36]. We hypothesized that the necessary reduction equivalents could be obtained from the pentose phosphate pathway (PPP) if a small amount of extra sugar was supplied in addition to d-GalA [37]. Since d-Xyl, l-Ara, and l-Rha are part of the pectin backbone and side chains, it is feasible that these monosaccharides help to alleviate the imbalance in reduction equivalents for *N. crassa* during pectin degradation. To test this hypothesis, we inoculated an overnight culture of WT, Δ*pdr*-*1*, and pdr-1-comp strains into a medium containing 2 mM d-GalA and 0.5 mM d-Xyl. After 3 days of growth, the WT and pdr-1-comp strains exhibited a significantly increased dry weight on the d-GalA plus d-Xyl medium as compared to growth on either d-GalA or d-Xyl alone (Fig. 1B). In contrast, the Δ*pdr*-*1* strain failed to accumulate additional biomass on the d-GalA plus d-Xyl medium, but instead performed similarly to biomass accumulation solely on d-Xyl. These results indicate that (1) PDR-1 plays a role during d-GalA catabolism in *N. crassa*, and (2) that the reduced growth on d-GalA in WT cells can be alleviated by adding additional monosaccharides.

### PDR-1 regulation affects HG degradation and pectin-related catabolism

**Fig. 2:**
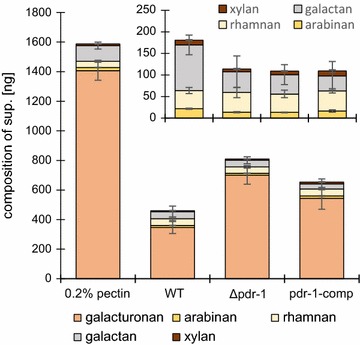
Pectin degradation profile of WT and Δ*pdr*-*1* supernatants. Residual culture supernatants of *N. crassa* WT, Δ*pdr*-*1*, and pdr-1-comp strains grown for 2 days on 0.2% pectin as well as the pectin medium itself were broken down into monosaccharides for a compositional analysis. The sugars were quantified by HPAEC-PAD. The *inlay diagram* shows a magnified version of arabinan, rhamnan, galactan, and xylan. *Error bars* represent standard deviation (*n* = 3)

The observed growth phenotypes cannot answer the question of whether PDR-1 regulates intracellular or extracellular processes, or both, and to what extent PDR-1 is involved in the regulation. To address this question, a compositional analysis of pectin before and after incubation with the WT, Δ*pdr*-*1* and pdr-1-comp strains was performed. Since the Δ*pdr*-*1* mutant shows only ~25% of WT biomass when directly inoculated on pectin medium, all strains were pregrown on medium containing 2% sucrose and were subsequently switched to pectin for an additional 2 days. Supernatants as well as the original pectin medium were hydrolyzed by a combined acid and enzymatic hydrolysis [38, 39] and the resulting monosaccharide composition was analyzed by HPAEC-PAD (Fig. 2). The results of this analysis showed only minor differences in the residual l-Ara, d-Xyl, and d-Gal contents between WT, pdr-1-comp, and Δ*pdr*-*1* strains. Only the d-GalA levels were different, with the WT strain having the least amount of residual d-GalA in the supernatant and the Δ*pdr*-*1* mutant having the highest. Intriguingly, l-Rha levels were virtually unchanged as compared to uninoculated pectin in all strains, indicating that either the RG-I backbone had not been exposed yet or that the rhamnogalacturonan lyase ASD-1 was not active under the (slightly acidic) conditions applied. Thus, the compositional analyses indicated that mostly the HG degradation machinery was impaired in the Δ*pdr*-*1* mutant.

### Determination of PDR-1-regulated genes by RNA-seq

The growth phenotypes observed in the Δ*pdr*-*1* mutant implied that multiple pectin-related metabolic processes are impaired. To assess the PDR-1 regulon, the transcriptional profile of *N. crassa* WT and Δ*pdr*-*1* strains was determined by RNA-seq after a 4 h incubation on medium with no carbon source (NoC), 2 mM l-Rha, or 1% citrus peel pectin. The RNA-seq data were used for differential expression (DEseq) analysis. Initially, the DEseq analysis of WT grown on pectin or L-Rha versus NoC showed that most of the genes predicted to be involved in pectin degradation were significantly upregulated (fold change ≥3) on pectin (Table 1). Next, all genes that were more than threefold upregulated in the WT or Δ*pdr*-*1* strain on either pectin or l-Rha in comparison to NoC were matched against each other by creating a Venn diagram (Additional file 1: Figure S3A, Additional file 2: Tables S1, S2). A custom catalogue based on the MIPS Functional Catalogue Database (FunCatDB) [40] with expanded categories for cell wall degradation-related genes was used for a follow-up enrichment analysis. Functional categories were determined for each group (bin) of the Venn diagram with more than 20 genes. Categories with a *p* value ≤5 × 10^−5^ were deemed as significantly enriched (Additional file 2: Tables S3, S4).

**Table 1:**
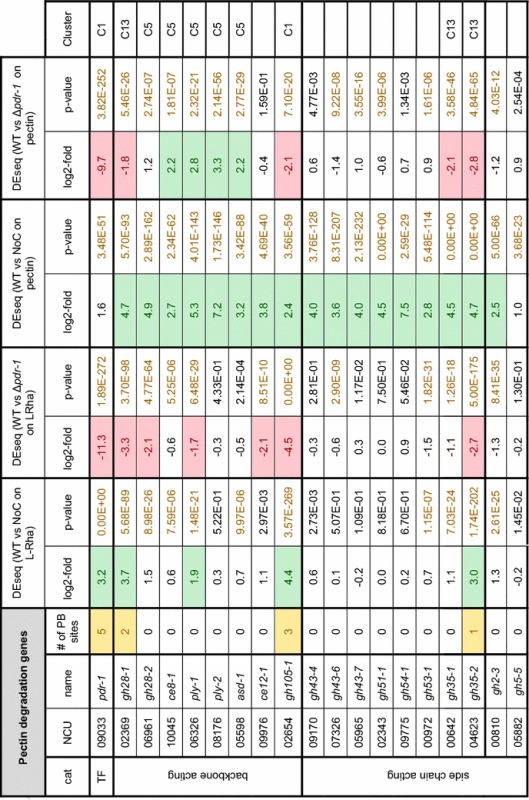

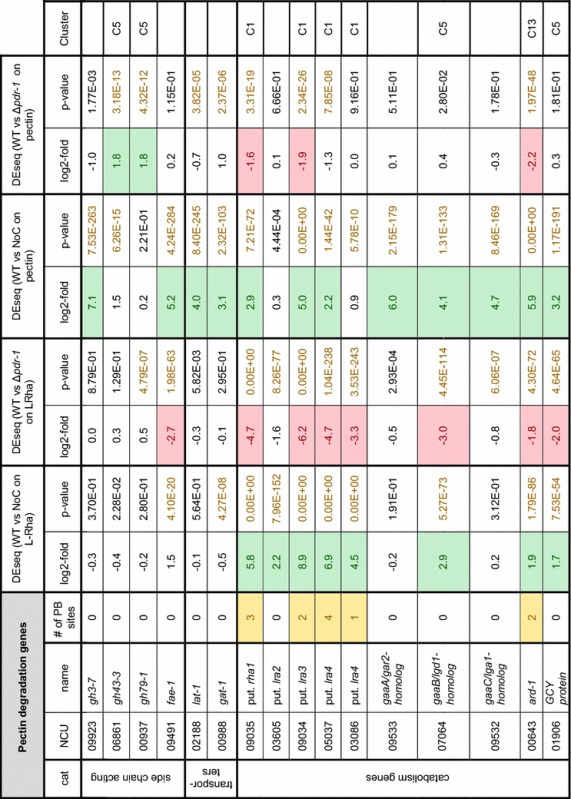
*Neurospora crassa* pectin degradation genes

**Fig. 3:**
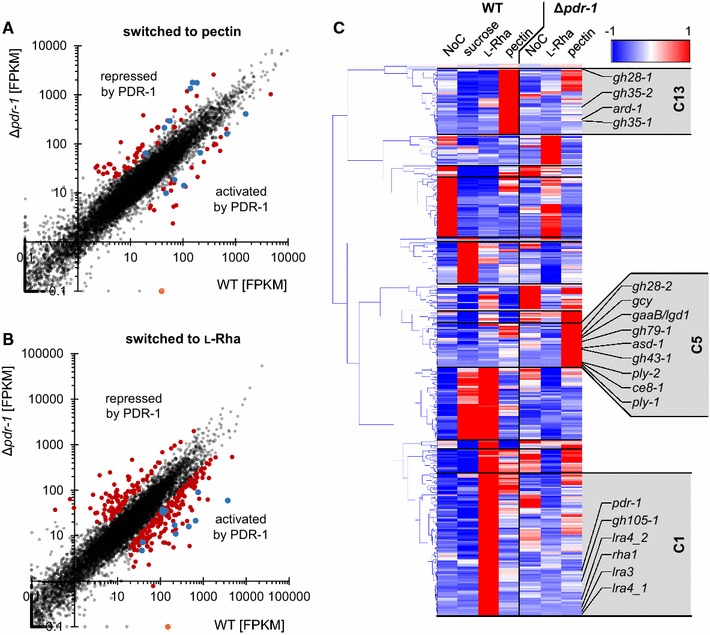
Scatterplots and clustering of RNA-seq data from WT and Δ*pdr*-*1*. Scatterplot diagram (**A**, **B**) axes are log_10_-scaled. **A** Full-genome expression profile of WT induced for 4 h on 1% pectin was plotted against the transcriptional response of the Δ*pdr*-*1* mutant induced on the same medium (*black dots*). Genes of Δ*pdr*-*1* that were threefold up- or downregulated to WT are marked with *red dots*. Genes that code for pectin degrading enzymes (according to Table 1) are represented by *blue colored dots*. The position of *pdr*-*1* is denoted with an *orange dot*. **B** Same as in **A**, but 2 mM l-Rha was used for induction. **C** 367 genes were either repressed or activated by PDR-1 and this subset was analyzed by hierarchical clustering. RNA-seq data of WT or Δ*pdr*-*1* after 4 h on either 2 mM l-Rha, 1% pectin, 2% sucrose (only WT), or no carbon source (NoC) were used. The position of pectinases and l-Rha/d-GalA catabolism genes are indicated next to their respective clusters (C1, C5, or C13). *lra4*_1 = NCU05037, *lra4*_2 = NCU03086

The number of PDR-1 consensus binding sites (PB sites) with a stringency score >0.6 are indicated. The fold change threshold for DEseq was 3 for l-Rha and pectin (log2-fold ≥1.58). *p* values <5 × 10^−5^ were considered significant. The Cluster number refers to Fig. 3

Among others, the functional categories for specific pectin catabolism (01.05.03.06.07.02) and/or hemicellulose/rhamnogalacturonan catabolism (01.05.03.06.07.03) were significantly enriched in the following bins: genes exclusively upregulated in WT on pectin and/or l-Rha (375 genes), the Δ*pdr*-*1* and WT strain on pectin (139 genes), as well as WT on both pectin and l-Rha together with the Δ*pdr*-*1* mutant on pectin only (31 genes). However, no significant enrichment for functional categories related to pectin degradation was found in the bins of genes solely upregulated in the Δ*pdr*-*1* strain on pectin (73 genes), l-Rha (332 genes), or under both conditions (56 genes), as well as the WT strain alone or together with Δ*pdr*-*1* on l-Rha (284 genes or 27 genes, respectively).

To determine which genes were differentially expressed in the Δ*pdr*-*1* strain compared to WT, a DEseq analysis of WT versus Δ*pdr*-*1* on 1% pectin was performed (Fig. 3A). Roughly, 99% (9186 of 9266 genes) of the genes showed a similar expression level in both strains on pectin. Intriguingly, of the remaining 80 genes, 49 genes exhibited an increased expression level in the Δ*pdr*-*1* mutant (Fig. 3A; red dots, repressed by PDR-1). Among these genes were all three lyases encoded in the *N. crassa* genome: the pectate lyases *ply*-*1* (NCU06326) and *ply*-*2* (NCU08176) and the rhamnogalacturonan lyase *asd*-*1* (NCU05598). In addition, the pectin methylesterase *ce8*-*1* (NCU10045), the β-glucuronidase *gh79*-*1* (NCU00937), and the putative β-1,4-xylosidase *gh43*-*3* (NCU06861) also showed increased expression levels in the Δ*pdr*-*1* mutant. Another 31 genes showed reduced expression levels in the Δ*pdr*-*1* mutant compared to the WT (Fig. 3A; red dots, activated by PDR-1), including the endo-polygalacturonase-encoding *gh28*-*1* (NCU02369) and genes encoding the unsaturated rhamnogalacturonyl hydrolase GH105-1 (NCU02654), the LRA3 homolog NCU09034, the β-galactosidases GH35-1 (NCU00642) and GH35-2 (NCU04623), and the l-arabinitol 4-dehydrogenase ARD-1 (NCU00643).

Overall, more genes showed a stronger misregulation between Δ*pdr*-*1* and WT strains on 2 mM l-Rha compared to the results on pectin (Fig. 3B). Applying a ±3-fold change threshold to these data, about 3% of the *N. crassa* genome (297 out of 9238 genes) showed an increased up- or downregulation. Of these genes, 91 showed higher expression levels in the Δ*pdr*-*1* mutant, although no pectinolytic gene was present in this gene set (Fig. 3B; red dots, repressed by PDR-1). The other 206 genes showed a higher expression in WT cells (Fig. 3B; red dots, activated by PDR-1). Among these genes were *gh28*-*1*, *gh28*-*2* (NCU06961), *gh35*-*2*, *gh105*-*1*, two putative *lra4* homologs (NCU03086, NCU05037), the putative *lra3* and *rha1* homologs (NCU09034 and NCU09035, respectively), the putative *gaaB/lgd1* homolog (NCU07064), *ard*-*1* (NCU00643), and the GCY protein-coding gene NCU01906. In particular, the failed induction of the endo-PG-encoding *gh28*-*1* and NCU07064, probably being responsible for the second step in d-GalA catabolism, might explain the observed growth phenotypes of the Δ*pdr*-*1* mutant on HG as well as on d-GalA.

To confirm the reliability of the RNA-seq data, the expression of several core pectinolytic genes was analyzed by quantitative real-time PCR (RT-qPCR) in the WT and Δ*pdr*-*1* strains after induction on pectin versus NoC, including *gh28*-*1*, *gh28*-*2*, *gh105*-*1*, *ce12*-*1*, *ce8*-*1*, *ply*-*1*, *ply*-*2*, and *asd*-*1* (Additional file 1: Figure S3B). A correlation analysis was performed between the RT-qPCR and RNA-seq data and the Pearson’s correlation coefficient (ρ) for WT and Δ*pdr*-*1* was 0.886 and 0.999, respectively, indicating a strong correlation of these datasets, and thus supporting the reliability of the RNA-seq results.

### Clustering of PDR-1-activated or PDR-1-repressed genes

Genes showing either a threefold up- or down-regulation in the DEseq analysis of WT versus Δ*pdr*-*1* strains on pectin or l-Rha versus NoC, respectively, were combined for hierarchical clustering. The FPKMs of these 367 genes in the WT and Δ*pdr*-*1* strains on NoC, 2% sucrose (WT only), 2 mM l-Rha, and 1% pectin were used for the clustering (Fig. 3C; Additional file 2: Table S5). The genes were distributed over 13 clusters with eight outliers. The clusters 1, 5, 12, and 13 contained genes with an increased expression specifically on one carbon source (cluster 1 and 13: on l-Rha or pectin in WT; cluster 5 and 12: on pectin or l-Rha in Δ*pdr*-*1*). Genes involved in pectin degradation and catabolism were only found in clusters 1, 5, and 13 (Table 1; Additional file 2: Table S5; Fig. 3C). Not surprisingly, most genes that showed increased expression levels in the Δ*pdr*-*1* strain on pectin grouped together in cluster 5 (*ply*-*1*, *ply*-*2*, *asd*-*1*, *ce8*-*1*, *ce79*-*1, gaaB/lgd1* homolog, *gh28*-*2*, gh43-1, NCU01906). Cluster 1 contained most genes predicted to be involved in the l-Rha catabolism (putative *rha1*, *lra3*, and *lra4* homologs), *gh105*-*1*, and the *pdr*-*1* gene itself, while cluster 13 comprised genes that increased in expression level in the WT on pectin (*gh28*-*1*, *gh35*-*1*, *gh35*-*2*, and *ard*-*1*).

### Differential regulation of HG acting enzymes by PDR-1

**Fig. 4:**
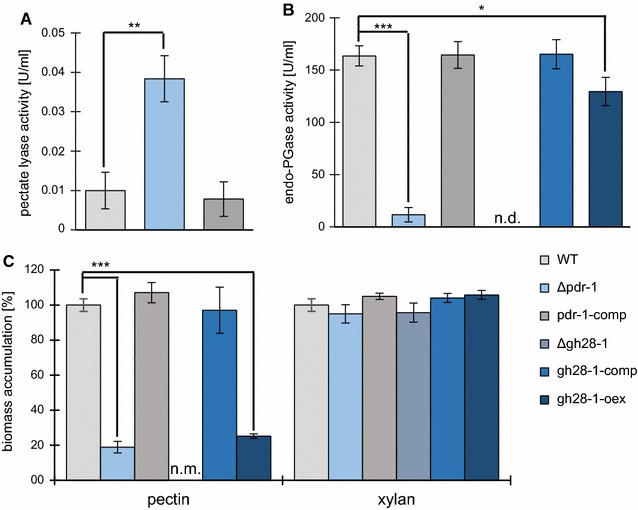
Enzymatic activity assays and biomass determination of Δ*pdr*-*1* and complementation constructs. Strains were grown for 3 days on either 1% pectin or 1% xylan. **A** Supernatants from the WT, Δ*pdr*-*1*, and pdr-1-comp strains were used to analyze pectate lyase activity. **B** endo-PGase activity of culture supernatants from the WT, Δ*pdr*-*1*, and pdr-1-comp strains (n.d. stands for none detected). **C** Biomass of the strains by dry weight. Biomass of Δ*gh28*-*1* on 1% pectin was not measurable (n.m.), since supernatant was highly viscous and could not be separated from mycelium. *Error bars* represent standard deviation (*n* = 3). Significance was determined by an independent two-sample *t*-test of WT against all other strains with *p < 0.05, **p < 0.01, and ***p < 0.001

The RNA-seq data showed a significantly weaker expression of the endo-PGase gene *gh28*-*1* in the Δ*pdr*-*1* strain on pectin, while the expression level of pectate lyases was increased (Fig. 3A; Table 1). To confirm whether the observed changes in expression of genes coding for HG-degradative enzymes are translated from the RNA to the protein level, the *N. crassa* WT, Δ*pdr*-*1,* and pdr-1-comp strains were grown on 1% pectin (or 1% xylan as control carbon source) and mycelial biomass and culture supernatants were taken for dry weight measurements and enzymatic activity assays, respectively, after 3 days of growth. When the pectate lyase activity in culture supernatants from WT, Δ*pdr*-*1*, and pdr-1-comp strains was compared, the Δ*pdr*-*1* strain showed significantly increased pectate lyase activity (Fig. 4A), consistent with the RNA-seq data.

For the analysis of endo-PGase activity, additionally the control strain Δ*gh28*-*1* was used. As expected, the *gh28*-*1* deletion strain showed no detectable enzymatic activity, while the activity of the WT and pdr-1-comp strains was similar (Fig. 4B). However, the Δ*pdr*-*1* strain showed significantly reduced endo-PGase activity, consistent with the RNA-seq results that showed a strongly reduced expression of *gh28*-*1* in this mutant.

### Restoration of endo-PGase activity is not sufficient to complement the Δ*pdr*-*1* phenotype

Since GH28-1 is the only endo-PGase in *N. crassa* and is important for growth on pectin [21], but was not properly induced in the Δ*pdr*-*1* strain (Figs. 3C, 4b), we hypothesized that overexpression of *gh28*-*1* might complement the pectin-deficient phenotype of the Δ*pdr*-*1* mutant. Thus, *gh28*-*1* was overexpressed in the Δ*gh28*-*1* background (strain gh28-1-comp) and the Δ*pdr*-*1* background (strain gh28-1-oex). Overexpression of *gh28*-*1* was confirmed by comparing both *gh28*-*1* overexpression strains to WT on pectin by RT-qPCR (Additional file 1: Figure S4A). Both strains showed ~3.5-fold elevated *gh28*-*1* expression levels as compared to the WT strain.

The extent of the complementation was determined by measurements of the dry weight and the endo-PGase activity in the culture supernatants of the used strains as compared to the WT strain and Δ*pdr*-*1* mutant grown on 1% pectin (Fig. 4B, C). The gh28-1-comp strain showed WT-like biomass accumulation and enzymatic activity, verifying that overexpression of *gh28*-*1* complemented the loss of the endo-PG. However, even though the endo-PGase activity in the gh28-1-oex strain increased to nearly WT levels (Fig. 4B), it still exhibited Δ*pdr*-*1* mutant-like growth on pectin (Fig. 4C). These results indicate that the reduced expression of *gh28*-*1* in the Δ*pdr*-*1* strain was not the sole reason for the observed growth phenotype of the Δ*pdr*-*1* mutant on pectin and suggests that also intracellular catabolic pathways might be affected.

### PDR-1 regulatory activity is associated with l-Rha

The expression profile of *pdr*-*1* on NoC, 2% sucrose, 2 mM l-Rha, and 1% pectin shows that *pdr*-*1* has its strongest expression level on l-Rha, and is least expressed on sucrose (Additional file 1: Figure S5A). Expression levels of *pdr*-*1* on NoC were increased compared to sucrose suggesting that *pdr*-*1* may be de-repressed in the absence of a carbon source. This de-repressed state could be achieved by autoinduction of PDR-1. However, it is known that some TFs need post-transcriptional or post-translational activation to become fully active [41, 42].

To test the hypothesis that PDR-1 needs to be post-transcriptionally activated for function, a strain was created that constitutively expresses *pdr*-*1* in the Δ*pdr*-*1* background (strain pdr-1-oex). The overexpression of *pdr*-*1* in this strain was confirmed by comparing its expression to the WT on pectin by RT-qPCR (Additional file 1: Figure S4A). The pdr-1-oex strain showed an ~33-fold increase in *pdr*-*1* expression compared to the WT. Next, the pdr-1-oex strain was grown for 3 days together with WT and the Δ*pdr*-*1* strain on xylan alone (as non-pectic control) or xylan with a regular supplementation of either 0.5 mM l-Rha or d-GalA. The biomass accumulation of these strains was determined by dry weight and their culture supernatants were used for endo-PGase activity assays. All strains were found to accumulate the same amount of biomass under all conditions (Additional file 1: Figure S5B), indicating that the low amount of inducing sugars (l-Rha or d-GalA) added to the medium was not sufficient for the generation of additional biomass.

**Fig. 5:**
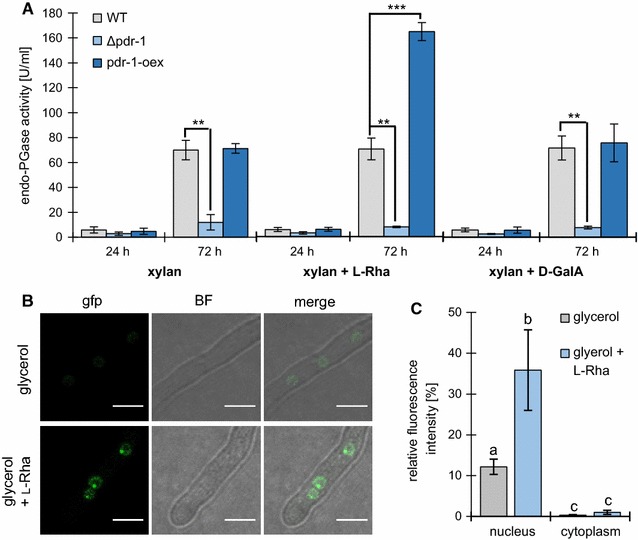
Endo-PGase activity of pdr-1-oex and subcellular localization of pdr-1-gfp in the presence of inducer molecules. **A** Strains were grown on 1% xylan plus 0.5 mM l-Rha or d-GalA or 1% xylan without any additives for 3 days. Endo-PGase activity was determined from culture supernatants taken after 24 or 72 h, respectively. *Error bars* represent standard deviation (*n* = 3). Significance was determined by an independent two-sample *t*-test of WT against all other strains with **p < 0.01 and ***p < 0.001. **B** The pdr-1-gfp strain was grown for 3 h on either 2% glycerol plus 2 mM l-Rha or 2% glycerol without any additives and fluorescence (gfp) as well as brightfield (BF) images were taken to determine the subcellular localization of PDR-1-GFP. *Scale bars* equals 5 µm. **C** Fluorescence intensity was measured for both culture conditions described in **B**. *Error bars* represent standard deviation (glycerol: *n* = 65; glycerol + l-Rha: *n* = 74). Significance was determined by analysis of variance (ANOVA) followed by a post hoc Tukey’s test. The *letters* above each *bar* indicate statistical significance; *bars* sharing the *same letter* show no significant difference, while *different letters* represent a significant mean difference of *p* < 0.001

Endo-PGase activity in the culture supernatants was measured at 24 h (before the addition of any monosaccharide) and at 72 h (after 48 h of incubation on xylan plus either l-Rha or d-GalA). Only weak endo-PGase activity could be measured for all strains and growth conditions after 24 h of incubation (Fig. 5A). After 72 h of growth, the WT and pdr-1-oex strains showed strongly increased enzymatic activity levels on xylan media, while the Δ*pdr*-*1* mutant showed a significantly weaker endo-PGase activity. However, the pdr-1-oex strain exhibited significantly increased activity levels specifically on xylan supplemented with l-Rha compared to WT on the same medium (Fig. 5), indicating that l-Rha is a specific inducer of this activity and that this is mediated by PDR-1.

In an additional set of experiments, the expression of *pdr*-*1* and a putative target gene, the *lra3* homolog NCU09034, was determined in the presence or absence of l-Rha by RT-qPCR. The WT and pdr-1-oex strains were grown for 48 h on xylan. Following this incubation time, the medium was supplemented with 2 mM l-Rha and the expression of both target genes compared against the non-induced (xylan only) control after 30 min (Additional file 1: Figure S4B). While the WT strain showed an ~12-fold increase in *pdr*-*1* expression, the expression of *pdr*-*1* in the pdr-1-oex strain was independent of l-Rha. Nevertheless, both strains showed a strong upregulation of the target gene NCU09034 in the presence of l-Rha (WT: ~27-fold, pdr-1-oex: ~90-fold). Since *pdr*-*1* expression in the pdr-1-oex strain did not change in the same time (Additional file 1: Figure S4B), the drastically increased NCU09034 expression is most likely due to a post-transcriptional activation of PDR-1. Together with the results of the endo-PGase assays, the RT-qPCR data indicate that PDR-1 activity is directly or indirectly dependent on the presence of l-Rha.

To determine whether the regulation of PDR-1 might include nuclear exclusion like demonstrated for GaaR [30], a GFP-tagged *pdr*-*1*-fusion construct driven by a constitutive promoter was transformed into *N. crassa* WT, creating the pdr-1-gfp strain. This strain was incubated for 3 h in medium containing either 2% glycerol or 2% glycerol plus 2 mM l-Rha as a carbon source. Even under non-inducing conditions (glycerol), a weak fluorescence signal was detectable in the nuclei (Fig. 5B). However, in medium containing l-Rha, the nuclear signal was found to be several times stronger (Fig. 5C) and a small, but strongly fluorescing dot was visible inside most nuclei (Fig. 5B). Since the expression of GFP-tagged PDR-1 should be unaltered due to the utilization of a constitutive promoter, this indicates that PDR-1 is not induced but recruited into the nucleus when an inducing molecule like l-Rha is present.

### PDR-1-regulated genes share a DNA-binding motif

**Fig. 6:**
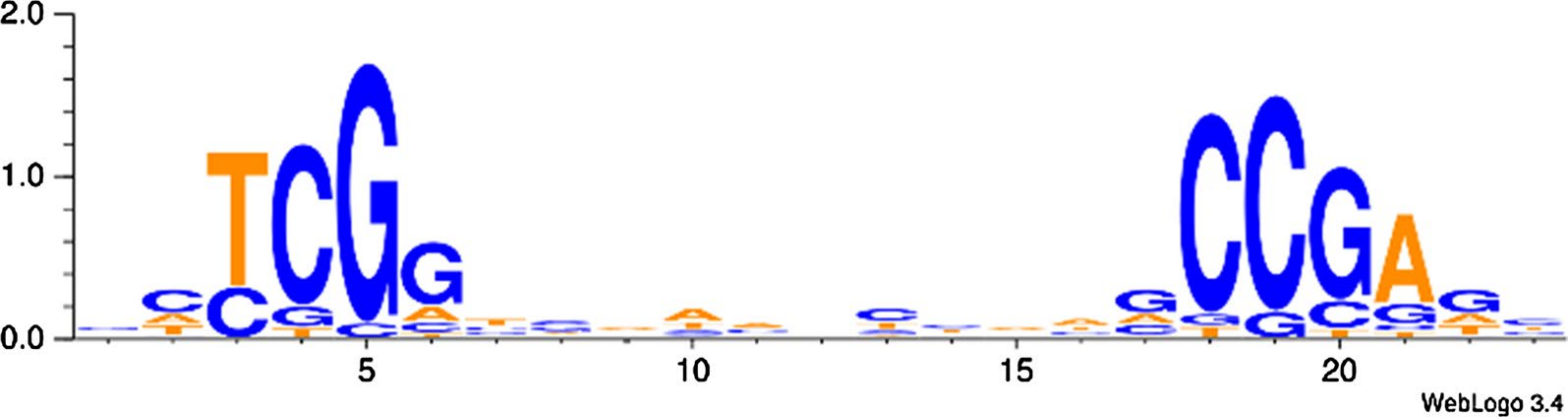
Putative consensus binding site of genes in the PDR-1 regulon. A palindromic core motif of TCGG with an 11 nucleotide long spacer region was identified as described in “Methods”

Pardo and Orejas [43] showed by EMSA analysis that the PDR-1 homolog RhaR has a palindromic core-binding motif of a CGG triplet on both sides of an 11 nucleotide long spacer region (CGG-X_11_-CCG). This DNA-binding site is typical for Gal4p-like TFs, which bind their recognition site as a homodimer [44, 45]. The corresponding consensus sequence for DNA binding of *N. crassa* PDR-1 was identified by Weirauch et al. [46] during a large-scale analysis of protein-binding microarrays, and it contained a highly conserved TCGG motif (available at: http://cisbp.ccbr.utoronto.ca/TFreport.php?searchTF=T161816_1.02). To increase the specificity of our query for a putative PDR-1-binding site, a search was performed in the promoter regions of five major pectinase genes regulated by PDR-1 (NCU09034, NCU09035, *gh28*-*1*, *gh105*-*1*, and *pdr*-*1* itself), in which we looked for the Gal4p-like inverted repeat with a TCGG motif, but also allowing for imperfect matches. This effort resulted in the consensus sequence as shown in Fig. 6. A full genome search query with this PDR-1-binding site returned 194 genes with a minimum of one binding site on the sense or anti-sense strand in the 1000 bp upstream region of the gene and a stringency score of 0.6 or greater (Additional file 2: Table S6; see “Methods”). The functional categories of these genes were determined, and the categories hemicellulose/pectin metabolism (01.05.03.06) and hemicellulose/pectin catabolism (01.05.03.06.07) were significantly enriched (*p* value below 5 × 10^−5^) (Additional file 2: Table S7). Among the 194 genes with a PDR-1-binding site were nine genes encoding pectinolytic enzymes that were regulated transcriptionally by PDR-1 (Table 1): *pdr*-*1*, *gh28*-*1*, *gh35*-*2*, and most genes of the l-Rha catabolism (encoding putative RHA1, LRA3, and LRA4 homologs).

### Towards the PDR-1 core regulon

A small set of 22 genes was obtained by matching all genes that contained a putative PDR-1 consensus binding site to the genes in the PDR-1-affected clusters 1, 5, 12, and 13 (Table 2). However, only genes in clusters 1 and 13 contained genes with a PDR-1-binding site including the nine pectinolytic ones mentioned above. Most of the putative l-Rha catabolism genes were present in this set (encoding the putative RHA1, LRA3, and LRA4 homologs), as well as the endo-PGase *gh28*-*1*, the β-galactosidases *gh35*-*2*, the l-arabinitol 4-dehydrogenase *ard*-*1*, the glycosyl hydrolase *gh105*-*1*, and *pdr*-*1*. The remaining 13 genes were found to encode the ribitol kinase *rok*-*1* (NCU01701), the allantoate permease *mfs*-*20* (NCU02653), the acylase (or putative gamma-glutamyltransferase) *acy*-*1* (NCU04130), the glycerol-3-phosphate *O*-acyltransferase *chol*-*6* (NCU05985), the quinate permease *mfs*-*19* (NCU06138), the endo-1,4-β-xylanase *gh11*-*2* (NCU07225), the fumarylacetoacetate hydrolase *fah*-*2* (NCU07506), the mitochondrial triosephosphate isomerase *emp*-*19* (NCU10106), a glyoxalase (NCU11253), and four hypothetical proteins (NCU01700, NCU01871, NCU08475, and NCU11278). Because all of these genes contain a putative PDR-1-binding site and showed a significantly reduced expression in the Δ*pdr*-*1* mutant, we conclude that these genes form the core PDR-1 regulon.

**Table 2:** Genes of the putative PDR-1 core regulon

ID	Name/annotation	# of PB sites	Cluster
NCU00643	*ard*-*1*	2	C13
NCU01700	Hypothetical protein	2	C1
NCU01701	*rok*-*1*	2	C1
NCU01871	Hypothetical protein	3	C1
NCU02369	*gh28*-*1*	2	C13
NCU02653	*mfs*-*20*	3	C1
NCU02654	*gh105*-*1*	3	C1
NCU03086	put. *lra4*	1	C1
NCU04130	*acy*-*1*	2	C1
NCU04623	*gh35*-*2*	1	C13
NCU05037	put*. lra4*	4	C1
NCU05985	*chol*-*6*	1	C1
NCU06138	*mfs*-*19*	1	C13
NCU07225	*gh11*-*2*	1	C13
NCU07506	*fah*-*2*	3	C1
NCU08475	Hypothetical protein	2	C1
NCU09033	*pdr*-*1*	5	C1
NCU09034	put. *lra3*	2	C1
NCU09035	put. *rha1*	3	C1
NCU10106	*emp*-*19*	2	C1
NCU11253	Glyoxalase	2	C1
NCU11278	Hypothetical protein	2	C1

PDR-1 consensus binding sites (PB sites) with a stringency score of more than 0.6 were used

## Discussion

Pectin is the most complex plant cell wall polysaccharide and requires a large number of enzymes to be efficiently broken down. This conceivably necessitates a similarly complex regulatory machinery for the coordination of the respective genes. When this study was initiated, no TF with a major growth defect mutant phenotype on pectin had been described in fungi. Using the genetic resources of *N. crassa*, we successfully identified *pdr*-*1* as the first such regulator, with the Δ*pdr*-*1* mutant showing a strongly impaired growth on citrus peel pectin. PDR-1 was found to be orthologous to the Zn(II)_2_Cys_6_ binuclear cluster TF TRC1 originally identified in *P. stipitis* [28]. The TRC1-like TFs are highly conserved in Ascomycetes and even several lineages of Basidiomycetes [43]. Functional information regarding this family is limited to TRC1 and its ortholog RhaR from the *Aspergilli* (Eurotiomycetes) [28, 29, 43]. In these organisms, TRC1/RhaR seem to be exclusively regulating the degradation of the pectic sub-domain RG-I or l-Rha catabolism, respectively. Interestingly, in other fungi, l-Rha has long been demonstrated to act as inducer for pectinolytic genes involved in the decomposition of the HG backbone. For instance, the bean anthracnose-causing phytopathogen *Colletotrichum lindemuthianum* secretes endo-PG in the presence of l-Ara and l-Rha [47]. With our finding that endo-PG is a core component of the PDR-1 regulon (demonstrated at the levels of endo-PG transcript, enzyme activity, and by the presence of two putative PDR-1-binding sites in the promoter region), this observation can now be explained on a molecular level. The situation in *P. stipitis* is different, since its genome does not encode any endo-PG or other pectinolytic activities [23]. The Eurotiomycetes (and most prominently the Aspergilli) on the other hand can be considered to represent the far end of the spectrum and have developed a highly expanded set of pectin catabolic genes including a number of additional TFs, such as GalX, GalR (only in *A. nidulans*), and AraR [48–50], that are not present in other Ascomycete species. Under these circumstances of increased division of labor, RhaR might have developed a more focused regulatory function.

We found PDR-1 to affect a broad functional spectrum of genes including factors involved in the degradation of the HG backbone, the RG-I backbone as well as the RG-I arabinogalactan side chains. Intriguingly, this effect is not limited to the secreted enzymes, but includes several (albeit not all) key factors of the respective monosaccharide catabolism. In case of HG decomposition, the inability of the Δ*pdr*-*1* strain to grow on both PGA and d-GalA alone (besides citrus peel pectin) thus seems a combination of the lack of induction of *gh28*-*1* as well as the l-galactonate dehydratase and LGD1 homolog NCU07064 (the second step in GalA catabolism). The exo-PG-encoding *gh28*-*2* seems somewhat affected by loss of PDR-1 as well, but its overall induction on pure l-Rha compared to pectin is very low. Moreover, we could not detect any putative PDR-1-binding site in the *gh28*-*2* promoter and do therefore not consider it part of the core PDR-1 regulon.

As reported for RhaR, the influence of PDR-1 on arabinogalactan side-chain degradation is limited [29]. Of 11 side chain-active enzymes that we found to be induced by l-Rha and/or pectin, only two β-galactosidases (*gh35*-*1* and *gh35*-*2*), a putative β-1,4-xylosidase (*gh43*-*3*), and a β-glucuronidase (*gh79*-*1*) were affected in the Δ*pdr*-*1* strain, thus reflecting the role of RhaR in *A. niger*. However, the fact that the l-arabinitol dehydrogenase *ard*-*1* is part of the PDR-1 regulon explains the additional strong growth defect of the deletion strain on both arabinan and l-Ara, which has not been reported for RhaR.

Regarding the RG-I backbone, we found the unsaturated rhamnogalacturonan hydrolase *gh105*-*1* to be a clear part of the PDR-1 regulon, which is intriguing, since the upstream acting rhamnogalacturonase *asd*-*1* (next to the pectate lyases *ply*-*1* and *ply*-*2*) was found to be regulated in an opposing manner. The rhamnogalacturonan acetylesterase *ce12*-*1* was also downregulated on l-Rha as shown in our RT-qPCR on pectin. This is the only observation that was not confirmed by RNA-seq analyses. However, we did not detect a putative PDR-1-binding site in the promoter region, and the overall expression level is extremely low regardless. Perhaps, *ce12*-*1* needs a more acetylated substrate to be efficiently induced, and for this reason does not appear in our scatterplot or clustering data (Fig. 3). In addition to these factors involved in RG-I backbone depolymerization, two genes that are a part of the core genomic cluster with *pdr*-*1*, namely the *rha1*-homolog NCU09035 and the *lra3*-homolog NCU09034, remain completely uninduced in the Δ*pdr*-*1* background. This can explain the inability of the Δ*pdr*-*1* strain to utilize l-Rha. Respectively, two and three putative PDR-1-binding sites were detected in the promoter regions of these genes, providing evidence for a direct regulation by the TF. The nature of the l-rhamnono-γ-lactonase (or LRA2-homolog) as well as the l-KDR aldolase (or LRA4-homolog) in *N. crassa* are unclear, as these factors are not clustered with the TF as it is the case for the Saccharomycotina TFs RhaR/TRC1 [28, 43]. However, a putative LRA2, NCU03605, as well as two possible LRA4 homologs, NCU05037 and NCU03086, were found to be strongly induced by l-Rha and downregulated in the Δ*pdr*-*1* strain compared to WT. The two putative LRA4-homologs additionally contain, respectively, one or four PDR-1-binding sites in their promoters. In summary, PDR-1 seems to control both extracellular depolymerization reactions as well as key steps in the catabolism of the liberated monosaccharides, and thus has a major effect on the degradation of pectin as a whole.

While PDR-1 appears to combine functions of all three recently reported pectinolytic TFs: Ara1 (from *M. oryzae*), GaaR (from *B. cinerea*), and RhaR (from the *Aspergilli*) [29–31], it is likely not the sole TF involved in regulating pectin catabolism in *N. crassa*. At least three observations support this conclusion: (1) The Δ*pdr*-*1* strain has a major growth defect on pectin, but is still able to accumulate between 20 and 30% of WT biomass. (2) Several genes encoding pectinolytic enzymes involved in HG backbone or RG-I side-chain degradation that are clearly induced by pectin do not appear to be l-Rha responsive or affected by loss of PDR-1. (3) De-repression of several pectinolytic genes in the absence of PDR-1 is almost exclusively seen on pectin and not l-Rha (cluster C5 in Fig. 3), and therefore when other signaling cascades (such as from d-GalA or l-Ara) are also active or even dominant. Moreover, none of the promoters of genes in cluster C5 have a detectable PDR-1-binding site. Taken together, this effect seems to be the result of an indirect action of PDR-1 in concert with one or several other TFs; potential candidates include the homologs of BcGaaR and MgAra1. In this respect, it is noteworthy that in *A. niger*, arabinan-degrading genes were also found to be more strongly expressed in Δ*rhaR* mutant than in the WT strain [29]. For these reasons, we conclude that PDR-1 has conserved functions in both activation and repression of transcription, likely as part of a larger regulatory network.

In *A. nidulans*, *rhaR* was reported to be neither auto-regulated nor carbon catabolite repressed. Based on this observation, the authors argued that RhaR is likely activated by post-transcriptional mechanisms in the presence of l-Rha [43]. In contrast, we found the expression of *pdr*-*1* to clearly respond to repressing and inducing conditions (Additional file 1: Figure S5A). These findings are in agreement with what was described for *rhaR* from *A. niger* [29]. It is possible that these contrasting results are due to the overall low expression values of *pdr*-*1*/*rhaR* typical for TFs that do not allow for semi-quantitative methods like the RT-PCR used by Pardo and Orejas [43] to pick up these changes accurately. Through constitutive expression of *pdr*-*1* in the pdr-1-oex strain, we aimed to uncouple post-transcriptional activation from transcriptional (auto)induction (Fig. 5). Two insights were generated from these experiments: (1) endo-PG activity even in the non-induced state suggests that a fraction of the PDR-1 pool is always active (or is de-repressed). Similar results were obtained on sucrose (data not shown). (2) Substantially higher endo-PG activity after addition of l-Rha, but not d-GalA, as well as strongly increased expression of NCU09034 in the pdr-1-oex strain indicated that PDR-1 is indeed post-transcriptionally—more likely post-translationally—regulated by its signaling molecule or a derivative thereof.

While some zinc binuclear cluster TFs, such as CLR-2 in *N. crassa*, are always in an active state and only regulated transcriptionally (in this case by CLR-1) [51, 52], activation by binding of a small molecular inducer or interaction with a metabolic intermediate is equally common [44]. In filamentous fungi, the pentoses d-Xyl and l-Ara or their respective catabolic intermediates have also been implicated as inducers for the hemicellulolytic pathways. As an effect of induction, XlnR was found to be reversibly hyper-phosphorylated in *Aspergillus* [42]. A similar effect has long been known for Gal4p in yeast [53]. Moreover, XYR1 in *Trichoderma* and XLR-1 in *N. crassa* have been engineered for constitutive activation, which could be a next logical step also for PDR-1 [54, 55].

What our data suggest is that the activated PDR-1 probably binds its responsive elements as a homodimer, since the DNA-binding site is arranged as an inverted repeat, typical for Gal4p-like TFs [44, 45]. However, to achieve specificity, we considered it unlikely that the originally described CGG-X_11_-CCG site [43], which is an exact replicate of the Gal4p responsive element, is the full recognition element. The extra T nucleotide we found was also present in the EMSA constructs used by Pardo and Orejas [43], in at least one half-site of the TCGG-X_11_-CGC motif found to be important for strong l-Rha-dependent induction of *lra3* in *Pichia pastoris* [56], and in the shorter binding motif identified by Weirauch et al. [46] (http://cisbp.ccbr.utoronto.ca/index.php). Therefore, we think it likely that the PDR-1/RhaR responsive element is represented by the TCGG-X_11_-CCGA motif.

Previous studies showed that subcellular localization and transport into the nucleus can play a crucial role in controlling how and when TFs can interact with their target gene. In *A. niger,* the xylanolytic and cellulolytic TF XlnR loses its regulatory function if it has certain mutations interfering with nuclear import [57]. The respective homolog in *T. reesei*, Xyr1, is also translocated into nuclei in the presence of xylose [58]. Similar translocation events were observed in *B. cinerea* for GaaR when d-GalA was used as carbon source [30]. In this study, we also observed a translocation process for *N. crassa* PDR-1 in the presence of l-Rha. PDR-1 contains one predicted NLS and NES region (Additional file 1: Figure S1) and since a constitutive promoter was used for expression in the pdr-1-gfp strain, the detected fluorescent signal was independent of transcriptional induction. Together with the observation of increased endo-PGase activity and NCU09034 expression after induction with l-Rha, it is feasible that (inactive) PDR-1 might be present in the cytoplasm and gets activated as well as translocated into nuclei in the presence of l-Rha.

A spot of increased fluorescence was visible inside most nuclei after induction, similar to what was observed by Zhang et al. for BcGaaR [30]. It is an intriguing possibility that these might represent PDR-1 bound to its promoter target sites. However, other explanations like the formation of regulatory protein complexes that include PDR-1 are also plausible [59, 60]. The mechanistic aspects of PDR-1/RhaR regulation, its mode of translocation, and the function of the observed nuclear bright spots are an intriguing topic for further study.

## Conclusions

Since PDR-1/RhaR is highly conserved across the genomes of Ascomycete and Basidiomycete species and present in many industrially and agriculturally important fungi, our findings contribute to a broader understanding of the pectinolytic processes in filamentous fungi. As mentioned above, pectinases are of value in the food and beverage industry and have additionally been shown to help lower the viscosity of feedstock fermentations by enhanced tissue maceration. In particular, endo-PG activity is required for the depolymerization of pectic gels. Based on our findings, PDR-1-mediated signaling is (1) not necessarily limited to the induction of rhamnogalacturonases, and (2) could be used to exclusively express endo-PG over exo-PG for these applications. It is furthermore noteworthy that for the expression of pectate lyases, the presence of PDR-1-like TFs should also be considered as overproduction of lyases was achieved by *pdr*-*1* deletion. Moreover, our results established that PDR-1/RhaR needs to be activated, and that expression of pectinases in pectin-free medium was possible by addition of a low amount of l-Rha in a strain overexpressing *pdr*-*1*.

In terms of agricultural applicability, endo-PG activity has been associated with pathogenicity and soft-rot-type disease symptoms in many filamentous fungi (e.g., [61] and reviewed in [62]), as well as with the mutual interaction of biocontrol species of *Trichoderma* with host plants [63]. The knowledge of the molecular regulator behind this expression might therefore prove valuable in the development of anti-fungal and biocontrol treatments.

## Methods

### Conserved protein domain search as well as determination of nuclear localization and export signals

The amino acid sequences for the *N. crassa* NCU09033, *A. niger* An13g00910 and *A. nidulans* AN5673, *P. stipitis* ABN68604 and *B. cinerea* Bcin09g00170 were retrieved from UniProt (http://www.uniprot.org/) as FASTA files. A phylogenetic tree of these proteins was generated by using the program Phylogen.fr [64, 65] with the following settings: alignment by MUSCLE excluding alignment curation by Gblocks and tree generation by PhyML.

NCU09033, An13g00910, AN5673, and ABN68604 were then analyzed using the conserved domain search tool provided by the NCBI (https://www.ncbi.nlm.nih.gov/Structure/cdd/wrpsb.cgi) [66–69]. Predicted Nuclear localization signals (NLS) in the amino acid sequences were determined using the NucPred (http://www.sbc.su.se/~maccallr/nucpred/) [70] and predicted nuclear export signals (NES) were detected with the NetNES 1.1 Server (http://www.cbs.dtu.dk/services/NetNES/) [71].

Protein sequence identity of full-length PDR-1 (NCU09033) to RhaR of *A. niger* (An13g00910) or *A. nidulans* (AN5673), as well as TRC1 of *P. stipitis* (ABN68604) was determined by comparing the retrieved amino acid sequences using BLASTp (https://blast.ncbi.nlm.nih.gov/Blast.cgi?PROGRAM=blastp&PAGE_TYPE=BlastSearch&LINK_LOC=blasthome) [72–74]. BLASTp was also used to compare the fungal transcription factor regulatory middle homology region of these four proteins.

### *Neurospora crassa* strains and growth conditions

The *N. crassa* WT strain and all deletion strains used in this work were obtained from the FGSC (http://www.fgsc.net, [75]; Additional file 2: Table S8). The strains were grown on 1× Vogel’s salts [76] and one of the following carbon sources (w/v): 1% arabinan (Megazyme, P-ARAB), 1% arabinogalactan (Megazyme, P-ARGAL), 2 mM l-arabinose (Sigma Aldrich, 10839), 1% cellulose (Avicel^®^ PH-101; Sigma Aldrich, 11365), 1% pectin (citrus peel, Sigma Aldrich, P9135), 1% polygalacturonic acid (PGA, Sigma Aldrich, 81325), 2 mM l-rhamnose (Sigma Aldrich, R3875), 2% sucrose (Sigma Aldrich, S7903), 1% xylan (beechwood, Sigma Aldrich, X4252), and 2 mM d-xylose (Sigma Aldrich, X3877). A medium containing 1× Vogel’s salts, 2 mM d-galacturonic acid (Sigma Aldrich, 48280), and 0.5 mM d-xylose was used for the growth experiment of *N. crassa* on d-galacturonic acid. The growth conditions are described further below.

The experiments regarding the pdr-1-oex strain were conducted on medium of 1× Vogel’s salts plus 1% (w/v) xylan. After 24 h of incubation at 25 °C, 200 rpm, and constant light, a solution containing 1× Vogel’s salts plus 0.5 mM l-Rha or d-GalA was added to the xylan medium two times a day for the remainder of the 3-day incubation period. Dry weight and endo-PGase activity were determined as explained in the respective method section.

### Biomass and enzymatic activity assays

The strains were used in triplicates and grown in 24-deep-well plates that were directly inoculated with 1 × 10^6^ conidia/ml in a volume of 3 ml medium. The incubation was conducted at 25 °C, 200 rpm, and constant light for 3 days (7 days in case of arabinogalactan and PGA) if not stated otherwise.

All strains grown on medium containing monosaccharides were pre-incubated for 16 h in medium containing 1× Vogel’s salts and 2% sucrose. After the pre-incubation, the strains were washed three times with 1× Vogel’s salts solution and transferred to the monosaccharide medium. The medium of each culture was switched two times a day during the incubation period.

To determine the biomass of the strains, the mycelial mass was dried for 16 h in aluminum pans at 105 °C and measured afterwards.

Endo-polygalacturonase (endo-PGase) activity of taken supernatants was measured with a ruthenium red assay as described by Ortiz et al. [77].

To determine the pectate lyase activity, a modified version of the pectate lyase activity assay from Megazyme was used (Megazyme, Ireland). Briefly, 200 µl of a 2.5 mg/ml polygalacturonic acid solution in 50 mM TRIS HCl buffer pH 8 solution was combined with 200 µl of a 50 mM TRIS HCl buffer pH 8 plus 1 mM CaCl_2_ solution. The solutions were briefly mixed at room temperature and 100 µl of culture supernatant was added to the mixture directly before measuring the absorbance over 10 min at 235 nm.

### Protein concentration

*Neurospora crassa* WT, Δ*pdr*-*1*, and pdr-1-comp were pregrown on 1× Vogel’s salts plus 2% (w/v) sucrose for 16 h at 25 °C, 200 rpm, and constant light. Following this incubation period, the strains were washed three times with 1× Vogel’s salts solution and afterwards transferred to a medium containing 1× Vogel’s salts and 0.2% (w/v) pectin. The strains were incubated for an additional 2 days and their supernatant was taken. The samples supernatants were 10-fold concentrated with a Vivaspin 6 centrifugal concentrator (Sigma Aldrich, Z614467) according to the manufacturers’ instructions. Protein concentration of the concentrated culture supernatants was measured with Roti^®^-Quant (Carl Roth, K015.1) as described by the manufacturer.

### Compositional analysis of pectin and culture supernatants

*Neurospora crassa* WT, Δ*pdr*-*1*, and pdr-1-comp were grown in triplicates and pre-incubated for 16 h on 1× Vogel’s salts plus 2% (w/v) sucrose, washed three times with 1× Vogel’s salt solution, and then transferred to a medium containing 1× Vogel’s salts and 0.2% (w/v) pectin. They were incubated for 2 days at 25 °C, 200 rpm, and constant light. Supernatants of the strains as well as the 0.2% pectin medium were taken and treated with a combined chemical and enzymatic hydrolysis as reported by Garna et al. [38, 39] with the following modified procedure. 500 µl of pectin solution or supernatant was combined with 500 µl of a 0.4 M TFA solution. The samples were incubated for 72 h at 80 °C. Afterwards, the pH was adjusted to 5 and the sample volume increased to 25 ml. Only 5 ml of the dilution were combined with 5 ml of a 500-fold diluted Viscozyme^®^ L (Sigma Aldrich, V2010) solution. The mixture was incubated for 24 h at 50 °C.

The released monosaccharides from the hydrolysis were analyzed by high-performance anion exchange chromatography with pulsed amperometric detection (HPAED-PAD) by using  an ICS-3000 instrument (Thermo Scientific, USA). The samples were injected into a 3 × 150 mm CarboPac PA20 column (Thermo Scientific) with a 3 × 30 mm guard column of the same material or a 3 × 250 mm CarboPac PA200 column (Thermo Scientific) with a 3 × 50 mm guard column of the same material. The samples were eluted from the CarboPac PA20 column at 30 °C using an isocratic mobile phase of 5 mM NaOH at 0.4 ml/min for 15 min. The elution of the samples from the CarboPac PA200 was also performed at 30 °C, but a gradient of 5–17 mM sodium acetate in 0.1 M NaOH over 12.5 min at 0.4 ml/min was used.

### Cloning of complementation and overexpression constructs

To create the *pdr*-*1* complementation strain (pdr-1-comp), the *pdr*-*1* gene as well as its promoter region and terminator region were cloned into the plasmid pCSR-knock-in, which contains 5′ and 3′ *csr*-*1* homology regions. The fragment was amplified from WT gDNA with the primers AT**GCGGCCGC**AGGCCGACGTGCCCTTCC and AT**GCGGCCGC**ATGCTTATGCCTATGCCTGCC, both primers contain a NotI restriction site (restriction sites in boldface). The finished construct was transformed into the Δ*pdr*-*1* strain by electrotransfection.

The *pdr*-*1* gDNA was inserted between NotI and PacI sites on the pTSL126B plasmid to create the *pdr*-*1* overexpression strain (pdr-1-oex) by using the primers AT**GCGGCCGC**ATGGCTGCTGCTGCTTCCG and GC**TTAATTAA**ATCGGATGCAAACCACCCTC. The plasmid contains the strong promoter of the glyceraldehyde-3-phosphate dehydrogenase (*gpd*) gene of *Myceliophthora thermophila* promoter and TrpC terminator and those regions are flanked by 5′ and 3′ *csr*-*1* homology regions for site-directed integration of the construct into the *N. crassa* genome. Similar to the *pdr*-*1* complementation strain, the pdr-1 overexpression construct was transformed into the Δ*pdr*-*1* strain by electrotransfection.

*Neurospora crassa* WT gDNA was amplified by PCR using the following primers ATTA**CCTGCAGG**ATGAAAATCACCGCCTCTCTTTTGC and ATCA**TTAATTAA**CTAACACCACGCGCCCGA to create a *gh28*-*1* (NCU02369) construct with SbfI and PacI restriction sites. This fragment was inserted in the plasmid pTSL126B between the respective restriction sites to create a *gh28*-*1* overexpression construct. The plasmid containing *gh28*-*1* was transformed into strains with a *gh28*-*1* (gh28-1-comp) or *pdr*-*1* deletion background (gh28-1-oex).

The pdr-1-gfp strain was created by amplifying the *pdr*-*1* gene from gDNA with the following primers: **CATCAACCAAATCTAG**ATGGCTGCTGCTGCTTCCGG and **CCTTAATTAACCCGGG**AAGAGTCGCCCCAGCGCCTTC (16 bp sequence overlaps to the pCCG-C-Gly-GFP plasmid in boldface). The plasmid pCCG-C-Gly-GFP, which contains 5′ and 3′ *his*-*3* homology regions, the constitutive promoter of the glucose-repressible gene-1 (*grg*-*1*, synonymously known as *ccg*-*1*), *adh*-*1* terminator and gfp sequence was amplified with the primers **CGCTGGGGCGACTCTT**CCCGGGTTAATTAAGGGCGGA and **AAGCAGCAGCAGCCAT**CTAGATTTGGTTGATGTGAGGGG (16 bp sequence overlaps to the *pdr*-*1* gene in boldface). The PCR products were digested over night with *Dpn*I, purified, and used for advanced quick assembly (AQUA) cloning, as described in [78]. The finished construct was transformed into the WT *his*-*3* ^−^ strain by electrotransfection.

### Media shift assays for RNA-seq and qPCR

Conidia obtained from 10-day-old pregrown cultures were used to inoculate 3 ml of 1× Vogel’s salts plus 2% (w/v) sucrose at 1 × 10^6^ cells/ml in 24-deep-well plates. The bottoms of the wells for each plate were initially scratched with a sharp needle to allow adherence and formation of mycelial mat. After 16 h of growth, mycelia were washed three times in 1× Vogel’s salts without added carbon and transferred to 1× Vogel’s salts with their respective new carbon source (as indicated). After 4 h of induction, mycelia were harvested over Whatman #1 filter paper and subsequently flash frozen in liquid nitrogen for storage at −80 °C. Total RNA was isolated as previously described [21].

### RNA-seq library prep

Stranded cDNA libraries were generated using the Illumina TruSeq Stranded RNA LT kit. mRNA was purified from 1 µg of total RNA using magnetic beads containing poly-T oligos. The purified mRNA was fragmented and reverse transcribed using random hexamers and SSII (Invitrogen) followed by second strand synthesis. The fragmented cDNA was treated with an end-pair, A-tailing, adapter ligation, and ten cycles of PCR.

The prepared library was then quantified using KAPA Biosystem’s next-generation sequencing library RT-qPCR kit and run on a Roche LightCycler 480 real-time quantitative PCR instrument. The quantified library was then multiplexed with other libraries, and the pool of libraries was then prepared for sequencing on the Illumina HiSeq sequencing platform utilizing a TruSeq paired-end cluster kit, v3, and Illumina’s cBot instrument to generate a clustered flowcell for sequencing. Sequencing of the flowcell was performed on the Illumina HiSeq 2000 sequencer using a TruSeq SBS sequencing kit, v3, following a 2 × 100 indexed run recipe.

The base calling of the libraries was performed with the Illumina CASAVA v1.8.2 package for the WT samples induced on sucrose, pectin and rhamnose, and the Illumina CASAVA v1.8.4 package for the rest of the samples. The raw reads were then evaluated for quality using both BBDuk (Geneious) and fastx toolkit (Hannonlab). The filtered reads were then mapped against *N. crassa* OR74A genome (v12) using Tophat 2.0.4 [79]. Transcript abundance was estimated with Cufflinks 2.0.2 [80] in fragments per kilobase of transcript per million mapped reads (FPKMs) using upper quartile normalization and mapping against reference isoforms from the Broad Institute. Tophat mapped reads were additionally counted by HTSeq 0.6.0 [81] to obtain raw counts. Differential expression analysis was performed on raw counts with DEseq 2 version 3.3 [82] using data from biological triplicates (WT or Δ*pdr*-*1* induced on 1× Vogel’s salts plus either no carbon source, 2 mM l-Rha, 2% sucrose, or 1% pectin (Δ*pdr*-*1* only)) or duplicates (WT induced on 1× Vogel’s salts plus 1% pectin).

### Quantitative real-time PCR

Quantitative real-time qPCR (RT-qPCR) was performed as described in [21]. The following genes were used for the correlation analysis of RT-qPCR to RNA-seq data: *asd*-*1*, *ce12*-*1*, *ce8*-*1*, *gh28*-*1*, *gh28*-*2*, *gh105*-*1*, *ply*-*1*, and *ply*-*2*. *N. crassa* WT and Δ*pdr*-*1* strains were incubated for 4 h on 1× Vogel’s salts plus 1% pectin to induce gene expression. Expression on 1× Vogel’s no carbon (NoC) was used as the reference sample.

Expression of *pdr*-*1* and *gh28*-*1* was determined by growing the WT, pdr-1-oex, gh28-1-comp, and gh28-1-oex strain on 1× Vogel’s salts plus 1% pectin for 48 h. Gene expression in WT was used as reference to calculate the relative expression of both target genes.

WT and pdr-1-oex were grown for 48 h on 1× Vogel’s salts with 1% xylan as carbon source. After this incubation period, 2 mM l-Rha was added to the medium and the strains were incubated for an additional 30 min. WT and pdr-1-oex incubated for 30 min without added l-Rha were used as controls. Gene expression of the strains incubated only on 1% xylan medium was used as reference to calculate the relative expression of both target genes.

Expression data of all RT-qPCR experiments were normalized to the endogenous control actin (NCU04173).

### Scatterplots of RNA-seq data

The RNA-seq data of WT and Δ*pdr*-*1* strains induced for 4 h on 1× Vogel’s salts plus either 1% pectin or 2 mM l-Rha were plotted against each other to create scatterplots. Genes with a threefold up- or down-regulation in the DEseq analyses were marked in the corresponding scatterplot as either “repressed by PDR-1” or “activated by PDR-1”, respectively. Genes below a minimum expression-threshold of 20 FPKMs for Δ*pdr*-*1* and WT were excluded. In addition, a log_2_-fold-change for the FPKMs of WT vs Δ*pdr*-*1* on both induction conditions was calculated and only genes with a log_2_-fold-change of either ±1.58 were marked, respectively. Samples for RNA-seq were prepared in triplicates [WT and Δ*pdr*-*1* on either no carbon source, 2 mM l-Rha, 2% sucrose or 1% pectin (only Δ*pdr*-*1*)] or duplicates (WT on 1% pectin).

### Hierarchical clustering of RNA-seq data

Hierarchical clustering of the averaged FPKMs of all RNA-seq library replicates was performed using the ‘Hierarchical Clustering Explorer’ v3.5 software (http://www.cs.umd.edu/hcil/multi-cluster/hce3.html) and the used settings were as described in [21].

### Promoter sequence studies

A modified version of the consensus sequence for PDR-1 binding identified by Weirauch et al. was used to identify genes with a PDR-1-binding site [46] (http://cisbp.ccbr.utoronto.ca/TFreport.php?searchTF=T161816_1.02). The sequence was extended by a palindromic core-binding motif of a TCGG quadruple on both sides of an 11 nucleotide long spacer region (TCGG-X_11_-CCGA), derived from EMSA analysis of Pardo and Orejas [43] and a manual extension of the site by analyzing the putative promoter-binding sites 1000 bp upstream of five major pectinase genes regulated by PDR-1 (NCU09034, NCU09035, *gh28*-*1*, *gh105*-*1*, and *pdr*-*1* itself).

The derived position weight matrix (PWM, Fig. 6) was used to search for potential binding sites in the promoter regions of *N. crassa* genes (defined as 1000 bp regions upstream of genes). The PWM contained only frequencies of each base at each position in the motif, so it was transformed into normalized—log likelihood form [83]: P_*k*,*j*_ = log_2_(M_*k*,*j*_/b_*k*_), where M_*k*,*j*_ is the frequency of base *k* at position *j* of motif, and b_*k*_ is the background frequency of *k* base in the *N. crassa* genome.

We searched only for potential binding sites with a stringency value >0.6 and the stringency value was defined as the ratio of motif score to the maximum possible score for the given PWM.

### Microscopic analysis

Conidia of pdr-1-gfp were inoculated on glass slides coated with a ~1-mm-thick layer of 2% Agar–Agar Kobe I (Carl Roth, 5210.2) dissolved in water. They were incubated for 3 h in medium containing 1× Vogel’s salts plus 2% glycerol (Carl Roth, 3783.1) for the non-induced condition or the same medium supplemented with 2 mM l-Rha for the induced condition. After the incubation period, the slides were covered with cover slips and GFP fluorescence images were made. The GFP fluorescence was imaged using a Leica TCS SP5 System (Leica Microsystems, Wetzlar, Germany) with laser excitation set to 488 nm. The LAS AF software was used to capture and analyze images.

ImageJ was used to measure fluorescence intensity of several nuclei and their surrounding cytoplasm on 6–10 images for each condition randomly. A background correction was performed by measuring several empty spaces on each image and subtracting the measured intensity from all other results. The resulting intensity measurements were processed in Microsoft Excel.

## Additional files

**Additional file 1: Figure S1.** Schematic depiction of phylogeny and conserved domains/signals in the *pdr*-*1* gene and its orthologs. The amino acid sequence of *N. crassa* PDR-1 (NCU09033), *A. niger* RhaR (An13g00910), *A. nidulans* RhaR (AN5673) and *P. stipitis* TRC1 (ABN68604) was used in a conserved domain search, as well as NLS and NES prediction. The phylogeny of these proteins was determined. *B. cinerea* GaaR (Bcin09g00170) constitutes the outgroup in the phylogenetic tree. GAL4 = GAL4-like Zn(II)_2_Cys_6_ (or C6 zinc) binuclear cluster DNA-binding domain, fungal TF MHR = fungal transcription factor regulatory middle homology region, green triangle = nuclear localization signal, red triangle = nuclear export signal. **Figure S2.** Growth phenotypes and protein secretion of *N. crassa* WT, Δ*pdr*-*1* and pdr-1-comp strains. (A) Observed growth phenotypes. Strains were grown on either 2 mM l-Rha, 2 mM d-Xyl, 1% pectin or 1% xylan. The cultures were incubated for 3 days. (B) Sucrose pregrown cultures were switched to pectin medium and the concentration of secreted protein was determined. Error bars represent standard deviation (*n* = 3). Significance was determined by an independent two-sample *t*-test of WT against Δ*pdr*-*1* or pdr-1-comp with *p < 0.05. **Figure S3.** Venn diagrams of DEseq results and correlation studies of RNA-seq to RT-qPCR data. Strains were pregrown for 16 h on 2% sucrose and then switched to an induction medium of either 1% pectin (pec) or 2 mM l-Rha for an additional 4 h. (A) Differential expression analysis (DEseq) was performed on the RNA-seq data. Genes of the WT and the Δ*pdr*-*1* strains that were threefold upregulated (left diagram; +) or downregulated (right diagram; −) were compared. Venn diagrams were created with: http://bioinformatics.psb.ugent.be/webtools/Venn/. WT on 1% pectin was used in biological duplicates; all other conditions were used in biological triplicates for the RNA-seq analysis. (B) Correlation analysis of RT-qPCR data to RNA-seq data. Axes are log_10_-scaled. The fold change expression of several key pectinase genes was determined by RT-qPCR in the WT and Δ*pdr*-*1* strain grown on 1% pectin and plotted against their respective RNA-seq data. The Pearson’s correlation coefficient (ρ) was determined. Two biological replicates were used for RT-qPCR except for *ply*-*2*, where only one was used. All biological replicates were analyzed as three technical replicates. **Figure S4.** Expression levels of *pdr*-*1*, *gh28*-*1* and *NCU09034* determined by RT-qPCR. (A) The strains were grown for 2 days on 1% pectin and the expression level of *pdr*-*1* in the pdr-1-oex strain or *gh28*-*1* in the gh28-1-oex and gh28-1-comp strain was determined. The WT strain was used as reference. (B) The WT and pdr-1-oex strain were incubated for 48 h on 1% xylan. 2 mM l-Rha was added and the strains were incubated for an additional 30 min before harvesting the RNA for RT-qPCR. Strains incubated for the same time but without l-Rha were used as controls. The expression of *pdr*-*1* and NCU09034 was determined for both strains. Three biological replicates were used, except for *pdr*-*1* in WT on pectin and NCU09034 in WT on xylan plus l-Rha, where only two were used. All biological replicates were analyzed as three technical replicates. Error bars represent standard deviation. Significance was determined by an independent two-sample *t*-test with **p < 0.01, ***p < 0.001. **Figure S5.** Expression profile of *pdr*-*1* and biomass accumulation of the pdr-1-oex strain. (A) Expression profile of *pdr*-1. After a 16 h pre-incubation on 2% sucrose, biomass of the WT strain was washed three times with 1× Vogel’s solution and transferred to a medium of either no carbon source (NoC), 2% sucrose, 2 mM l-Rha or 1% pectin. The strain was incubated for an additional 4 h. Error bars represent standard deviation (*n* = 2 for 1% pectin, *n* = 3 for NoC, 2% sucrose and 2 mM l-Rha). (B) Determination of accumulated biomass. Strains were grown on 1% xylan with 0.5 mM l-Rha or d-GalA. A medium containing 1% xylan was used as control. Biomass was determined by dry weight. Error bars represent standard deviation (*n* = 3).

**Additional file 2.** Supplemental tables S1 – S8. Tables S1 and S2 contain the Venn diagrams of genes that were either 3-fold up- (S1) or down-regulated (S2) in the Δ*pdr-1* strain compared to the WT. The tables S3 and S4 show the FUNCAT analyses corresponding to the respective Venn diagrams. Table S5 contains the result of the clustering of genes that were either 3-fold up- or down-regulated in the WT and Δ*pdr-1* strains. In the tables S6 and S7, the results of the in silico PDR-1 binding motif studies (S6) and the corresponding FUNCAT analysis (S7) are presented. Table S8 contains a list of all *N. crassa* genes used in this work.
