# Supplementary material for: The transcription factor PDR-1 is a multi-functional regulator and key component of pectin deconstruction and catabolism in Neurospora crassa
Source: Biotechnol Biofuels. 2017 Jun 12;10:149. doi: 10.1186/s13068-017-0807-z (PMC5469009; doi:10.1186/s13068-017-0807-z)
Supplement: Supplementary file 1 — Additional file 1: Figure S1. Schematic depiction of phylogeny and conserved domains/signals in the pdr-1 gene and its orthologs. The amino acid sequence of N. crassa PDR-1 (NCU09033), A. niger RhaR (An13g00910), A. nidulans RhaR (AN5673) and P. stipitis TRC1 (ABN68604) was used in a conserved domain search, as well as NLS and NES prediction. The phylogeny of these proteins was determined. B. cinerea GaaR (Bcin09g00170) constitutes the outgroup in the phylogenetic tree. GAL4 = GAL4-like Zn(II)2Cys6 (or C6 zinc) binuclear cluster DNA-binding domain, fungal TF MHR = fungal transcription factor regulatory middle homology region, green triangle = nuclear localization signal, red triangle = nuclear export signal. Figure S2. Growth phenotypes and protein secretion of N. crassa WT, Δpdr-1 and pdr-1-comp strains. (A) Observed growth phenotypes. Strains were grown on either 2 mM l-Rha, 2 mM d-Xyl, 1% pectin or 1% xylan. The cultures were incubated for 3 days. (B) Sucrose pregrown cultures were switched to pectin medium and the concentration of secreted protein was determined. Error bars represent standard deviation (n = 3). Significance was determined by an independent two-sample t-test of WT against Δpdr-1 or pdr-1-comp with *p < 0.05. Figure S3. Venn diagrams of DEseq results and correlation studies of RNA-seq to RT-qPCR data. Strains were pregrown for 16 h on 2% sucrose and then switched to an induction medium of either 1% pectin (pec) or 2 mM l-Rha for an additional 4 h. (A) Differential expression analysis (DEseq) was performed on the RNA-seq data. Genes of the WT and the Δpdr-1 strains that were threefold upregulated (left diagram; +) or downregulated (right diagram; −) were compared. Venn diagrams were created with: http://bioinformatics.psb.ugent.be/webtools/Venn/. WT on 1% pectin was used in biological duplicates; all other conditions were used in biological triplicates for the RNA-seq analysis. (B) Correlation analysis of RT-qPCR data to RNA-seq data. Axes are log1 [file 13068_2017_807_MOESM1_ESM.pptx]

## Slide 1
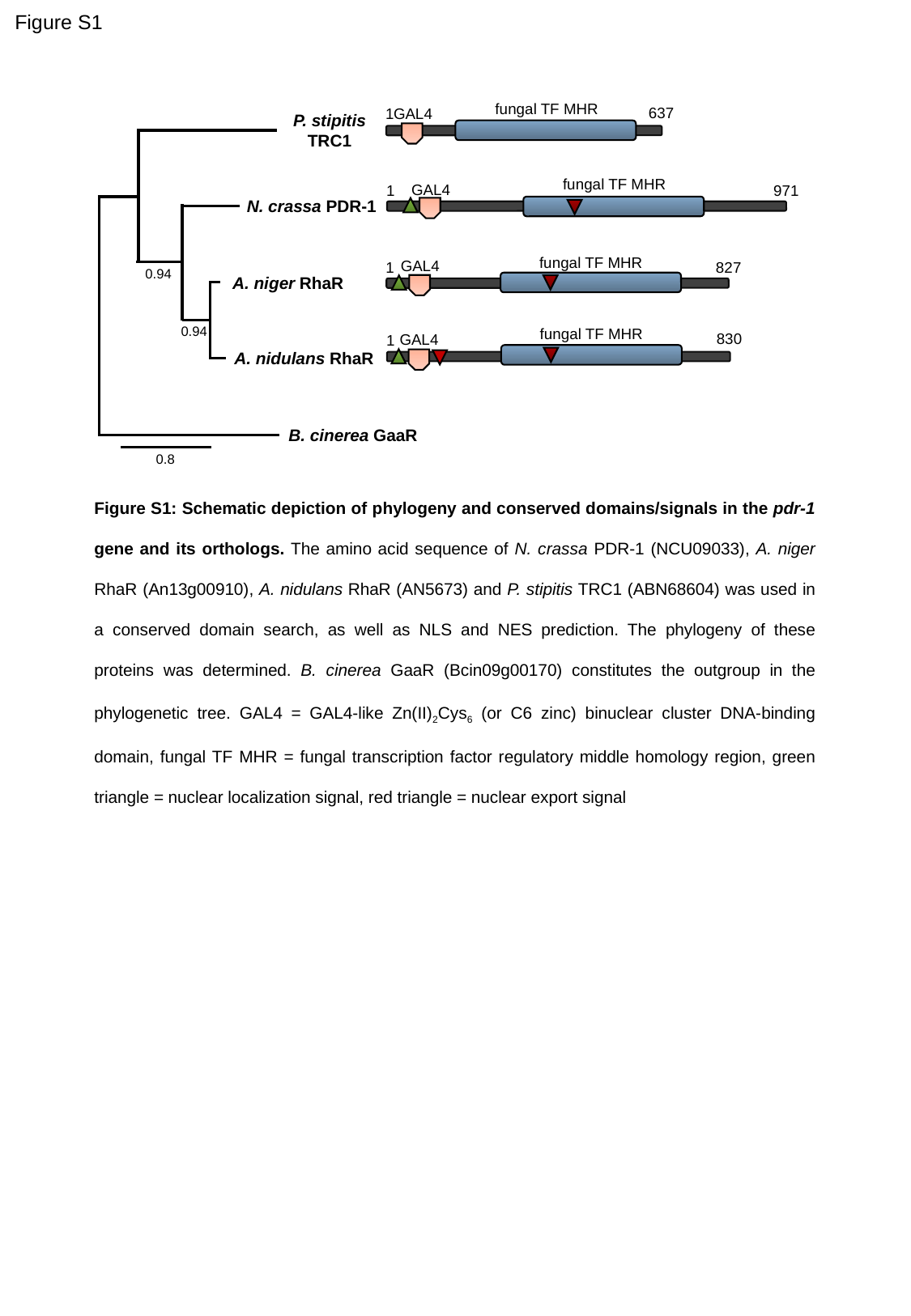

Figure S1
fungal TF MHR
637
GAL4
1
P. stipitis TRC1
fungal TF MHR
GAL4
1
971
N. crassa PDR-1
fungal TF MHR
GAL4
827
1
A. niger RhaR
fungal TF MHR
830
GAL4
1
A. nidulans RhaR
B. cinerea GaaR
0.94
0.94
0.8
Figure S1: Schematic depiction of phylogeny and conserved domains/signals in the pdr-1 gene and its orthologs. The amino acid sequence of N. crassa PDR-1 (NCU09033), A. niger RhaR (An13g00910), A. nidulans RhaR (AN5673) and P. stipitis TRC1 (ABN68604) was used in a conserved domain search, as well as NLS and NES prediction. The phylogeny of these proteins was determined. B. cinerea GaaR (Bcin09g00170) constitutes the outgroup in the phylogenetic tree. GAL4 = GAL4-like Zn(II)2Cys6 (or C6 zinc) binuclear cluster DNA-binding domain, fungal TF MHR = fungal transcription factor regulatory middle homology region, green triangle = nuclear localization signal, red triangle = nuclear export signal

## Slide 2
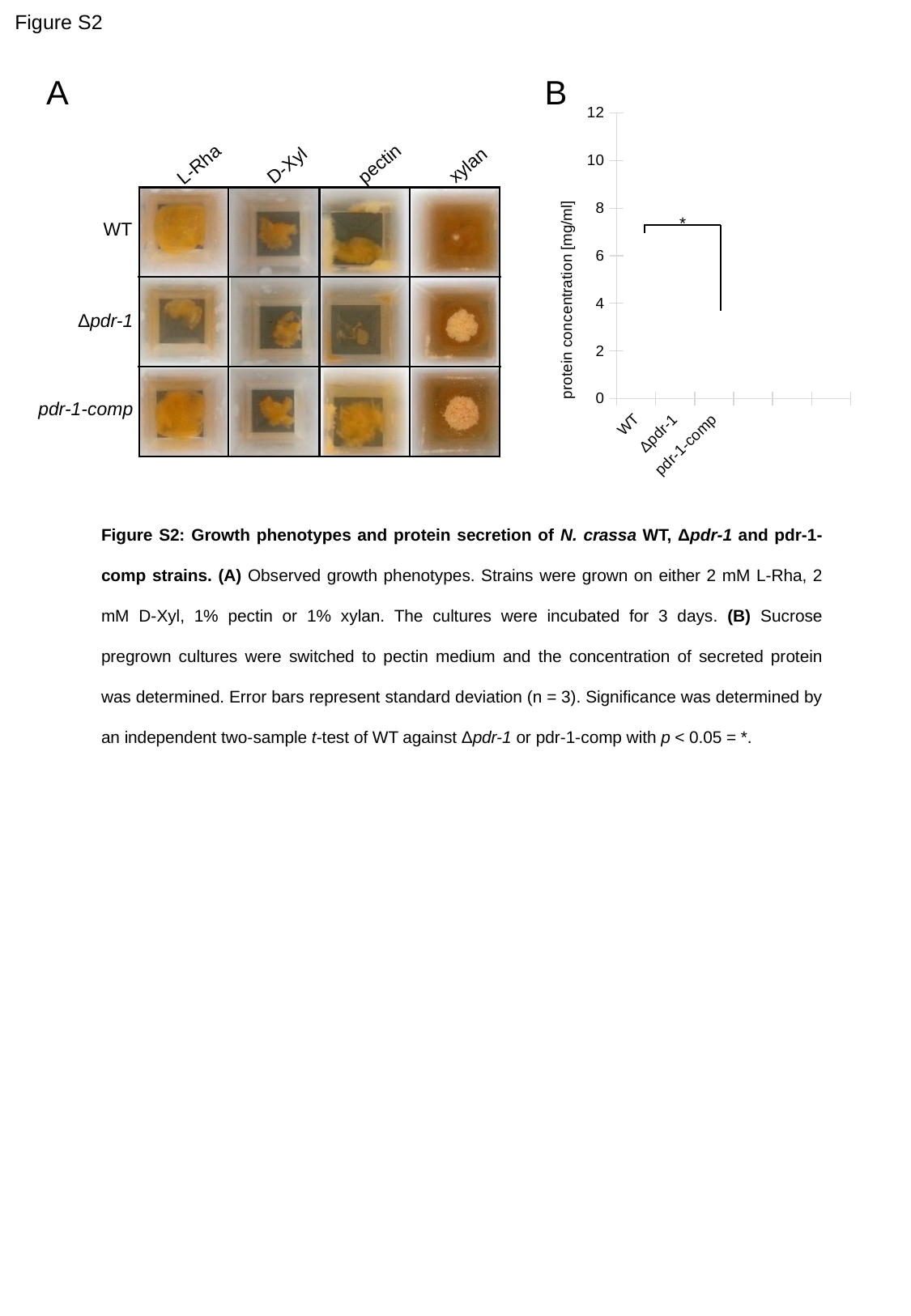

Figure S2
A
d-Xyl
l-Rha
xylan
pectin
WT
Δpdr-1
pdr-1-comp
B
### Chart
| Category | 0.2% pectin, vivaspin concentrated |
|---|---|
| WT | 5.66521717430889 |
| Δpdr-1 | 2.928984015920888 |
| pdr-1-comp | 7.824638293445974 |*
Figure S2: Growth phenotypes and protein secretion of N. crassa WT, Δpdr-1 and pdr-1-comp strains. (A) Observed growth phenotypes. Strains were grown on either 2 mM l-Rha, 2 mM d-Xyl, 1% pectin or 1% xylan. The cultures were incubated for 3 days. (B) Sucrose pregrown cultures were switched to pectin medium and the concentration of secreted protein was determined. Error bars represent standard deviation (n = 3). Significance was determined by an independent two-sample t-test of WT against Δpdr-1 or pdr-1-comp with p < 0.05 = *.

## Slide 3
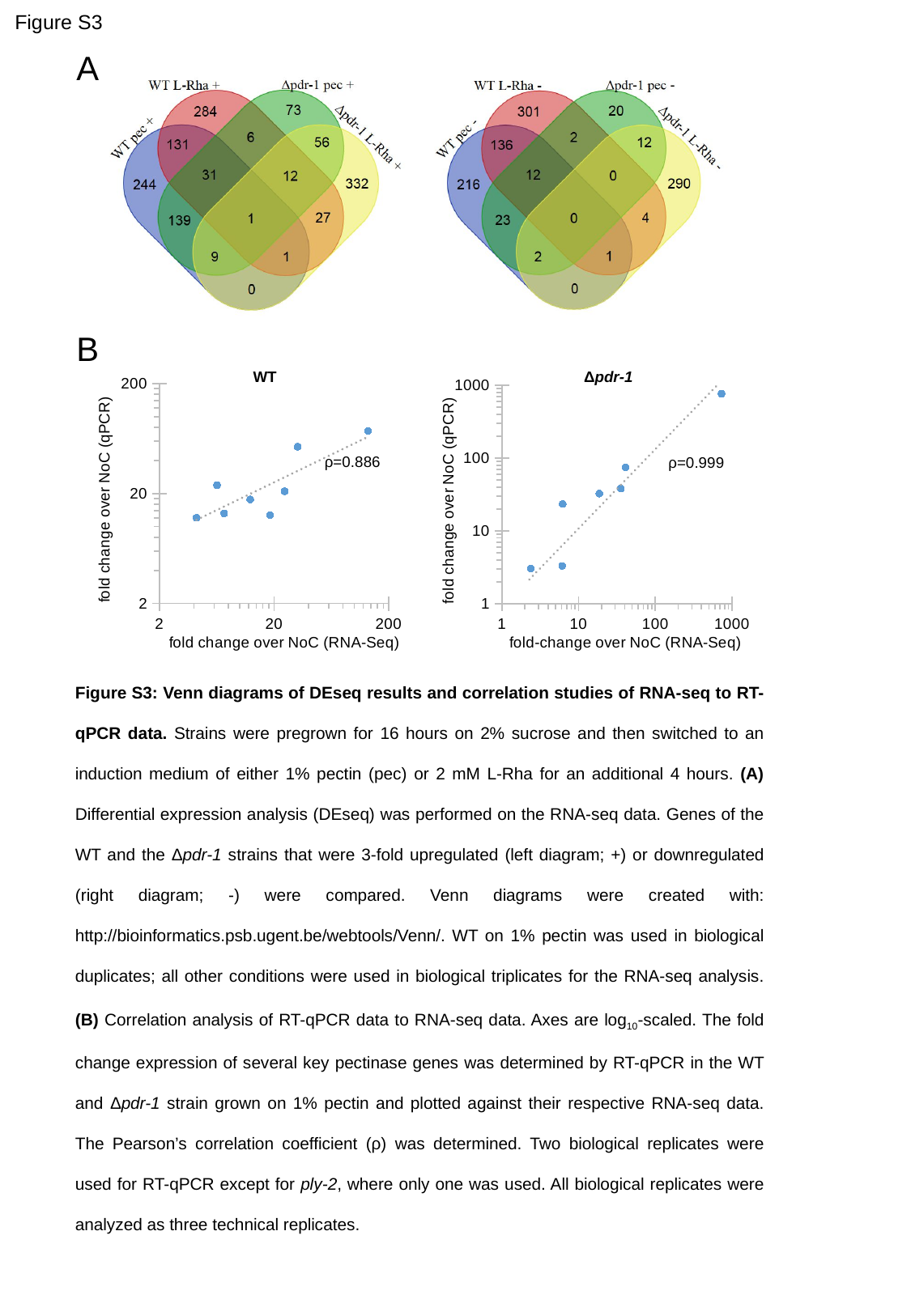

Figure S3
A
B
WT
Δpdr-1
### Chart
| Category | WT |
|---|---|
### Chart
| Category | Δpdr-1 |
|---|---|ρ=0.886
ρ=0.999
Figure S3: Venn diagrams of DEseq results and correlation studies of RNA-seq to RT-qPCR data. Strains were pregrown for 16 hours on 2% sucrose and then switched to an induction medium of either 1% pectin (pec) or 2 mM l-Rha for an additional 4 hours. (A) Differential expression analysis (DEseq) was performed on the RNA-seq data. Genes of the WT and the Δpdr-1 strains that were 3-fold upregulated (left diagram; +) or downregulated (right diagram; -) were compared. Venn diagrams were created with: http://bioinformatics.psb.ugent.be/webtools/Venn/. WT on 1% pectin was used in biological duplicates; all other conditions were used in biological triplicates for the RNA-seq analysis. (B) Correlation analysis of RT-qPCR data to RNA-seq data. Axes are log10-scaled. The fold change expression of several key pectinase genes was determined by RT-qPCR in the WT and Δpdr-1 strain grown on 1% pectin and plotted against their respective RNA-seq data. The Pearson’s correlation coefficient (ρ) was determined. Two biological replicates were used for RT-qPCR except for ply-2, where only one was used. All biological replicates were analyzed as three technical replicates.

## Slide 4
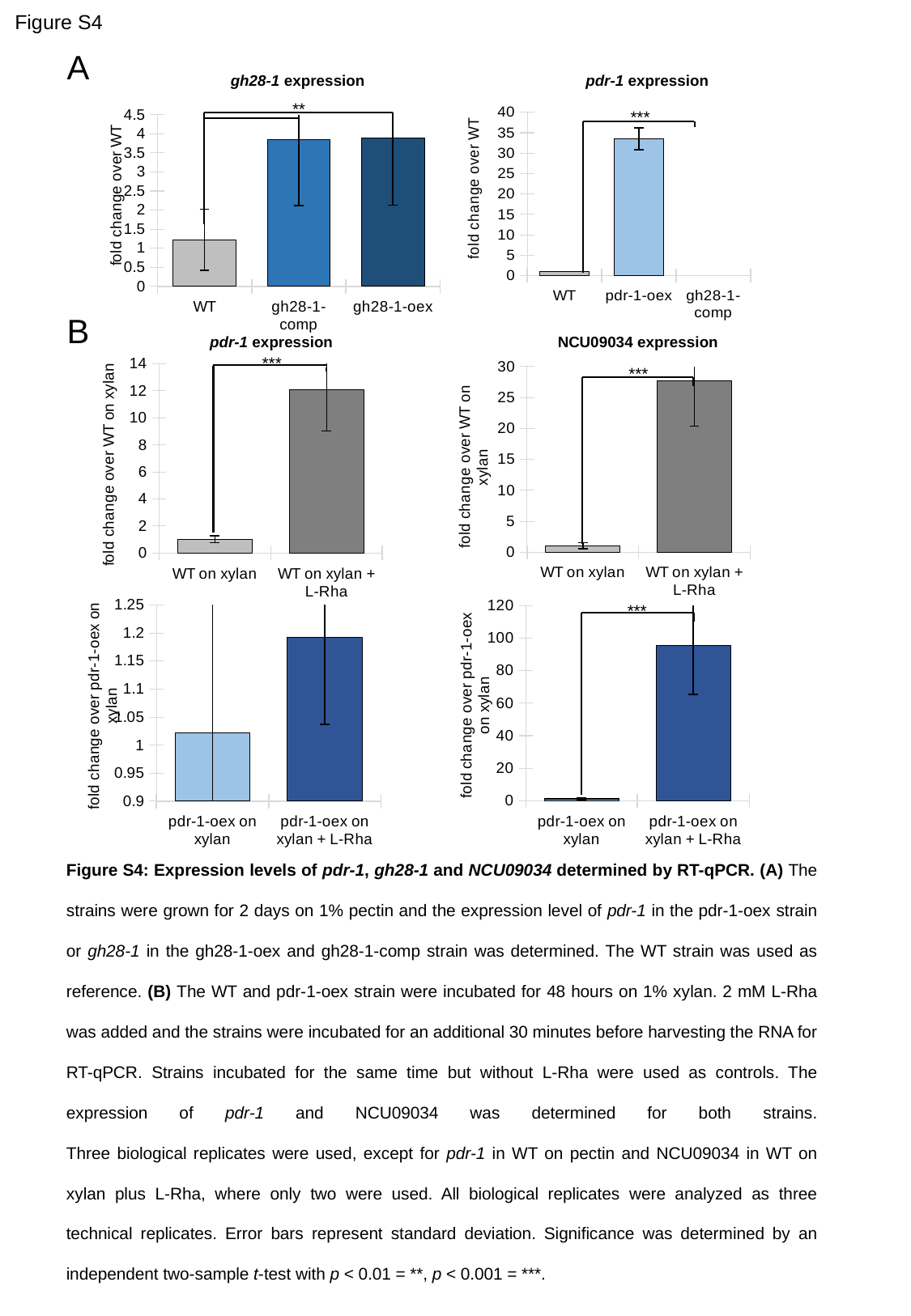

Figure S4
A
pdr-1 expression
gh28-1 expression
**
### Chart
| Category | gh28-1 |
|---|---|
| WT | 1.2190655858092976 |
| gh28-1-comp | 3.845905660934683 |
| gh28-1-oex | 3.8958476830931983 |
[unsupported chart]
***
B
NCU09034 expression
pdr-1 expression
***
### Chart
| Category | pdr-1 |
|---|---|
| WT on xylan | 1.0273953402381173 |
| WT on xylan + L-Rha | 12.089729560581159 |
### Chart
| Category | NCU09034 |
|---|---|
| WT on xylan | 1.091194664608457 |
| WT on xylan + L-Rha | 27.63017766596673 |***
### Chart
| Category |
|---|
### Chart
| Category | NCU09034 |
|---|---|
| pdr-1-oex on xylan | 1.177037976817968 |
| pdr-1-oex on xylan + L-Rha | 95.47057769958353 |***
### Chart
| Category | pdr-1 |
|---|---|
| pdr-1-oex on xylan | 1.0214623395338045 |
| pdr-1-oex on xylan + L-Rha | 1.1924374684507004 |Figure S4: Expression levels of pdr-1, gh28-1 and NCU09034 determined by RT-qPCR. (A) The strains were grown for 2 days on 1% pectin and the expression level of pdr-1 in the pdr-1-oex strain or gh28-1 in the gh28-1-oex and gh28-1-comp strain was determined. The WT strain was used as reference. (B) The WT and pdr-1-oex strain were incubated for 48 hours on 1% xylan. 2 mM l-Rha was added and the strains were incubated for an additional 30 minutes before harvesting the RNA for RT-qPCR. Strains incubated for the same time but without l-Rha were used as controls. The expression of pdr-1 and NCU09034 was determined for both strains.
Three biological replicates were used, except for pdr-1 in WT on pectin and NCU09034 in WT on xylan plus l-Rha, where only two were used. All biological replicates were analyzed as three technical replicates. Error bars represent standard deviation. Significance was determined by an independent two-sample t-test with p < 0.01 = **, p < 0.001 = ***.

## Slide 5
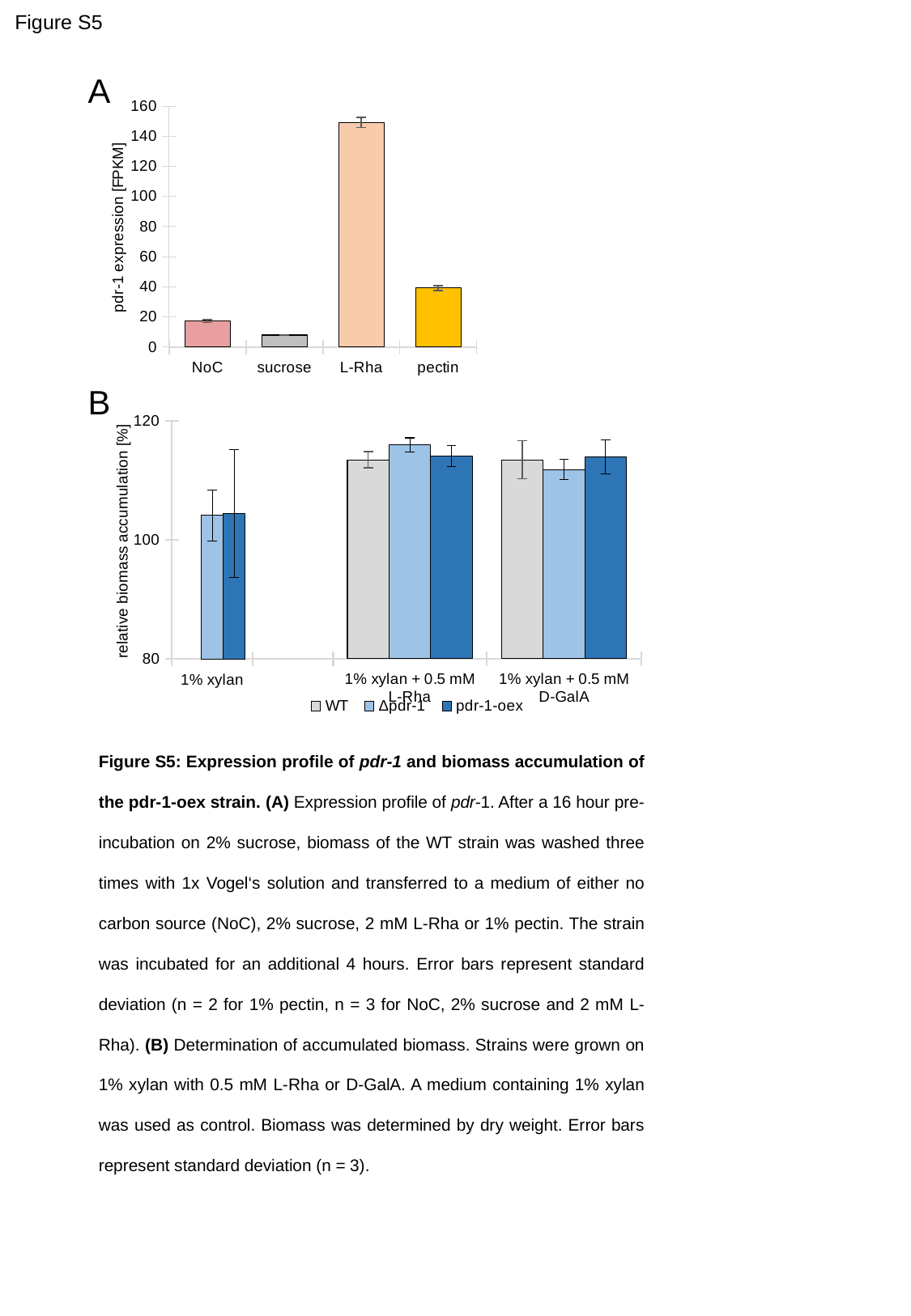

Figure S5
A
### Chart
| Category | pdr-1 |
|---|---|
| NoC | 17.110933333333335 |
| sucrose | 7.920133333333333 |
| L-Rha | 149.15833333333333 |
| pectin | 39.1636 |B
### Chart
| Category | WT | Δpdr-1 | pdr-1-oex |
|---|---|---|---|
| 1% xylan | 100.0 | 104.13223140495859 | 104.40771349862263 |
### Chart
| Category | WT | Δpdr-1 | pdr-1-oex |
|---|---|---|---|
| 1% xylan + 0.5 mM L-Rha | 100.0 | 107.48663101604278 | 101.87165775401066 |
| 1% xylan + 0.5 mM D-GalA | 100.0 | 95.11568123393316 | 101.28534704370179 |Figure S5: Expression profile of pdr-1 and biomass accumulation of the pdr-1-oex strain. (A) Expression profile of pdr-1. After a 16 hour pre-incubation on 2% sucrose, biomass of the WT strain was washed three times with 1x Vogel‘s solution and transferred to a medium of either no carbon source (NoC), 2% sucrose, 2 mM l-Rha or 1% pectin. The strain was incubated for an additional 4 hours. Error bars represent standard deviation (n = 2 for 1% pectin, n = 3 for NoC, 2% sucrose and 2 mM l-Rha). (B) Determination of accumulated biomass. Strains were grown on 1% xylan with 0.5 mM l-Rha or d-GalA. A medium containing 1% xylan was used as control. Biomass was determined by dry weight. Error bars represent standard deviation (n = 3).
